# Infrared Irradiation‐Assisted Green Approach for Pd‐Catalyzed Buchwald–Hartwig Amination

**DOI:** 10.1002/chem.202500557

**Published:** 2025-04-16

**Authors:** Ambra Maria Fiore, Riccardo Ciciriello, Davide Blasi, Pietro Cotugno, Angela Punzi, Gianluca Maria Farinola

**Affiliations:** ^1^ Dipartimento di Chimica Università degli Studi di Bari Aldo Moro Via Edoardo Orabona 4 Bari 70126 Italy

**Keywords:** Buchwald–Hartwig amination, C–N bond formation, green solvents, infrared irradiation, quasi‐solvent‐free conditions

## Abstract

We report a novel and green approach for Pd‐catalyzed Buchwald–Hartwig amination, assisted by Infrared (IR) irradiation under quasi‐solvent‐free conditions, in a nonanhydrous environment, and without the exclusion of air. The C–N coupling reactions, performed with a stoichiometric amount of cyclopentyl methyl ether, proceed with moderate‐to‐good yields using aryl iodides and bromides bearing both electron‐donating and electron‐withdrawing groups, along with a variety of secondary and primary amines.

## Graphical abstract

For the first time, infrared (IR) irradiation is demonstrated as an effective energy source, able to assist the Pd‐catalyzed Buchwald–Hartwig amination. The C–N coupling reactions were performed under quasi‐solvent‐free conditions, in nonanhydrous conditions and without the exclusion of air, using aryl iodides and bromides functionalized with both electron‐donating and electron‐withdrawing groups, along with a variety of secondary and primary amines.

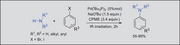

## 1 Introduction

The Green Chemistry Protocol aims to develop sustainable methods in chemistry by eliminating or reducing hazardous reactants and wastes, following the 12 principles proposed by Anastas and Warner.^[^ ^1^ ^]^ This goal can be achieved through the careful design and optimization of chemical products and processes. In this context, the selection and judicious use of reaction solvents are crucial, as they are one of the main contributors to the environmental impact of a chemical process. Therefore, it is no surprise that several principles of Green Chemistry offer effective solutions to this challenge. To prevent waste generation (principle 1), the use of reaction solvents, which are often the largest contributor to chemical waste by weight, should be eliminated or significantly reduced. If the use of solvent is necessary, preference should be given to solvents that are nontoxic for humans and not harmful for the environment (principle 5), nonhazardous to minimize the risk of chemical accidents (principle 12), and preferably derived from renewable sources (principle 7). Obviously, the use of innocuous and environmentally friendly solvents and the application of solvent‐free or quasi‐solvent‐free protocols (utilizing only stoichiometric amounts of solvent) represent cleaner and more sustainable alternatives to traditional solution‐based organic synthesis. These approaches mitigate the risks associated with toxic or hazardous solvents while reducing reaction waste.^[^ ^2^ ^]^ Design for energy efficiency (principle 6) is also important in order to develop sustainable protocols: energy requirements should be minimized for a favorable environmental and economical impact. To meet this target, the use of highly efficient activating reaction modes is required. Microwave irradiation^[^ ^3^ ^]^ and mechanical milling^[^ ^4^ ^]^ have been successfully used as alternative activating reaction modes to replace thermal heating, enabling shortened reaction times, and increased reaction yields. A cheaper alternative to these activating reaction modes, often requiring specific and expensive instruments, is infrared (IR) irradiation. In addition to transferring heat directly by radiation, IR irradiation can also excite molecular vibrational levels, both mechanisms making it a highly efficient activating mode in various chemical processes.^[^ ^5^ ^]^ In particular, the IR region of the electromagnetic spectrum is divided into three parts: shortwave infrared, also called near‐infrared (NIR), ranging from 0.76 to 2 µm; medium‐wave infrared, also called middle‐infrared (MIR), ranging from 2 to 4 µm; and long‐wave infrared, also called far‐infrared (FIR), ranging from 4 to 1000 µm. Emission in each of these three wavelength ranges requires the use of specific commercial infrared lamps.^[^ ^5b^ ^]^ Among them, NIR emitters (incandescent light bulbs equipped with a tungsten filament sealed in a quartz envelope with a halogen gas) are particularly notable for their rapid response time (less than 1 second after switch‐on) and efficient energy use, with 90% of the applied energy converted into infrared heat. This efficiency enables the achievement of high temperatures, significantly reducing the reaction time.^[^ ^5a^ ^]^ IR irradiation have been used as an efficient activation source in various chemical processes, including the selective extraction of natural products (mainly FIR‐assisted) and a variety of organic reactions, such as condensation reactions, oxidation reactions, and synthesis of heterocyclic compounds (mainly NIR‐ and MIR‐assisted).^[^ ^5^, ^6^ ^]^ Due to its efficiency as well as its instrumental cost‐effectiveness, IR activation represents a powerful tool for the development of sustainable synthetic procedures. Despite its great potential, only a few examples of palladium‐catalyzed coupling reactions promoted by NIR irradiation have been reported so far,^[^ ^7^ ^]^ including Mizoroki‐Heck,^[^ ^7a–c^ ^]^ Suzuki‐Miyaura,^[^ ^7c,d^ ^]^ or direct arylation^[^ ^7e^ ^]^ reactions.

The palladium‐catalyzed Buchwald–Hartwig amination represents one of the most powerful and versatile approaches for the formation of C(sp^2^)‐N bonds with wide applications in various research areas including drug discovery and materials science, in both academic and industrial processes.^[^ ^8^ ^]^ After the development of the first general procedure in 1995,^[^ ^9^ ^]^ subsequent efforts mainly focused on the improvement of the catalytic system by combination of metal pre‐catalysts with highly active phosphine or *N*‐heterocyclic carbene ligands.^[^ ^8a^ ^]^ Recently, the growing interest in sustainable chemical processes has led to the development of green protocols for the Buchwald–Hartwig reaction. In fact, although Buchwald–Hartwig aminations are commonly carried out in volatile and harmful organic solvents (e.g., toluene, DME, THF), environment‐friendly synthetic approaches based on the use of green solvents^[^ ^10^ ^]^ or solvent‐free conditions^[^ ^11^ ^]^ have been also reported. Moreover, nonconventional activating reaction modes, such as microwave irradiation^[^ ^11a^ ^]^ and mechanical milling,^[^ ^11c,d^ ^]^ have been employed to assist efficient Buchwald–Hartwig aminations, whereas protocols using IR irradiation have not been explored so far.

As part of our studies on development of sustainable Pd‐catalyzed protocols,^[^ ^7^, ^12^ ^]^ we report herein a Pd‐catalyzed Buchwald–Hartwig amination carried out (i) in a stoichiometric amount of cyclopentyl methyl ether (CPME), (ii) in nonanhydrous conditions and without the exclusion of air, and (iii) using IR irradiation as an activating reaction mode. CPME is well known to be an eco‐friendly ethereal solvent, produced through a 100 % atom‐economical reaction, and widely used to lower the environmental impact of fine‐chemical synthesis.^[^ ^13^ ^]^ Indeed, CPME possesses valuable properties, including low toxicity, low peroxide formation, stability towards both strong acids and bases, and high boiling point, making it less hazardous than other ethereal solvents.

## 2 Results and Discussion

In our preliminary investigations we have selected the reaction of aryl halides **1a‐c** with *N*‐methylaniline **2a** yielding to **3a** as the model reaction (Table 1). The reactions for the optimization of the experimental conditions were carried out using an excess of aryl halide (unless specified, **1**/**2a** ratio of 1.5/1), a commercially available Pd catalyst (unless specified, 5 mol%), and a base (1.5 equiv.) in CPME (3.4 equiv.) under IR irradiation for 2 hours. A very simple experimental setup was used, consisting of a glass reactor, a Carius tube in which all the reagents were charged in air, mounted beside an IR lamp that primarily emitted in the NIR region, with maximum intensity at approximately 1.2 µm (Figure S1). Initially, we performed the Buchwald–Hartwig amination of **1a** with **2a** using Cs_2_CO_3_ as the base in the presence of Pd(OPiv)_2_/PPh_3_ (entry 1) or Pd(OAc)_2_/PPh_3_ (entry 2) as the catalytic system. In both these experimental conditions the formation of the expected product was not observed. Subsequent reactions were carried out using a stronger and more soluble base, such as sodium *tert*‐butoxide, while evaluating different catalytic systems, as shown in entries 3–9. We found that the Buchwald–Hartwig aminations occurred in moderate‐to‐good conversion (40‐70%) using Pd(OAc)_2_ in the presence of phosphine ligands such as triphenylphosphine, tris(*o*‐methoxyphenyl)phosphine, or S‐Phos (entries 3–5), while low‐to‐moderate conversions were detected in the presence of Pd_2_(dba)_3_ (11%, entry 6) and Pd(PPh_3_)_2_Cl_2_ (54%, entry 7). Notably, the reaction conversion raised to 80% using Pd(PPh_3_)_4_ (entry 8) and to 100% using Pd(^t^Bu_3_P)_2_ (99% isolated yield, entry 9). Under these conditions, reducing the reaction time from 2 hours to 1 hour (entry 10) resulted in a modest decrease in conversion (from 100% to 88%). On the other hand, lowering the catalyst loading from 5 mol% to 2 mol% (entry 11) led to a substantial drop in conversion (from 100% to 40%). Under the optimized conditions defined in the entry 9, we examined the role of the aryl halide: using less reactive bromide instead of iodide still resulted in full conversion (entry 12), whereas the use of chloride (entry 13) significantly reduced the reaction conversion from 100% to 42%. Also decreasing the excess of aryl bromide from 1.5 equivalents to 1.2 equivalents (entry 14) reduced the reaction conversion from 100% to 84%. Finally, the model reaction was carried out under conventional thermal heating using an oil bath pre‐warmed at 110 °C that is approximately the internal reaction temperature measured under IR irradiation: a full conversion was achieved, but the coupling product **3a** was isolated in a lower yield (85%, entry 15).

**Table 1:** Optimization of the reaction conditions.^[a]^

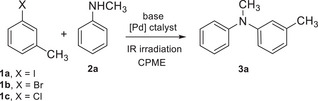						
Entry	ArX	Catalyst	Ligand [mol%]	Base	Conv^[b^ ^]^	Yield^[c]^
**1**	**1a**	Pd(OPiv)_2_	PPh_3_ (5)	Cs_2_CO_3_	0%	‐
**2**	**1a**	Pd(OAc)_2_	PPh_3_ (5)	Cs_2_CO_3_	0%	‐
**3**	**1a**	Pd(OAc)_2_	PPh_3_ (5)	NaO^t^Bu	57%	‐
**4**	**1a**	Pd(OAc)_2_	P(*o*‐MeOC_6_H_4_)_3_ (10)	NaO^t^Bu	40%	‐
**5**	**1a**	Pd(OAc)_2_	S‐Phos (10)	NaO^t^Bu	70%	‐
**6**	**1a**	Pd_2_(dba)_3_	‐	NaO^t^Bu	11%	‐
**7**	**1a**	Pd(PPh_3_)_2_Cl_2_	‐	NaO^t^Bu	54%	‐
**8**	**1a**	Pd(PPh_3_)_4_	‐	NaO^t^Bu	80%	‐
**9**	**1a**	**Pd(^t^Bu_3_P)_2_**	‐	**NaO^t^Bu**	**100%**	**99%**
**10** ^[d]^	**1a**	Pd(^t^Bu_3_P)_2_	‐	NaO^t^Bu	88%	‐
**11** ^[e]^	**1a**	Pd(^t^Bu_3_P)_2_	‐	NaO^t^Bu	40%	‐
**12**	**1b**	**Pd(^t^Bu_3_P)_2_**	‐	**NaO^t^Bu**	**100%**	**96%**
**13**	**1c**	Pd(^t^Bu_3_P)_2_	‐	NaO^t^Bu	42%	‐
**14** ^[f^ ^]^	**1b**	Pd(^t^Bu_3_P)_2_	‐	NaO^t^Bu	84%	‐
**15** ^[g]^	**1b**	Pd(^t^Bu_3_P)_2_	‐	NaO^t^Bu	100%	85%

^[a]^ Unless specified otherwise, reactions were carried out as follows: **1** (1.5 equiv.), **2a** (1 equiv.), Pd catalyst (5 mol%), base (1.5 equiv.) in CPME (3.4 equiv.) under IR irradiation for 2 hours.

^[b]^ Conversion determined by GC analysis on the crude product.

^[c]^ Yields on isolated products (column chromatography).

^[d]^ As entry 9, after 1 hour.

^[e]^ As entry 9, in the presence of 2 mol% of Pd(^t^Bu_3_P)_2_.

^[f]^ As entry 12, using 1.2 equiv. of **1b**.

^[g]^ As entry 12, under conventional thermal heating in a pre‐warmed (110 °C) oil bath for 2 hours (110 °C is approximately the reaction temperature measured under IR irradiation by an internal thermometer).

**Scheme 1:**
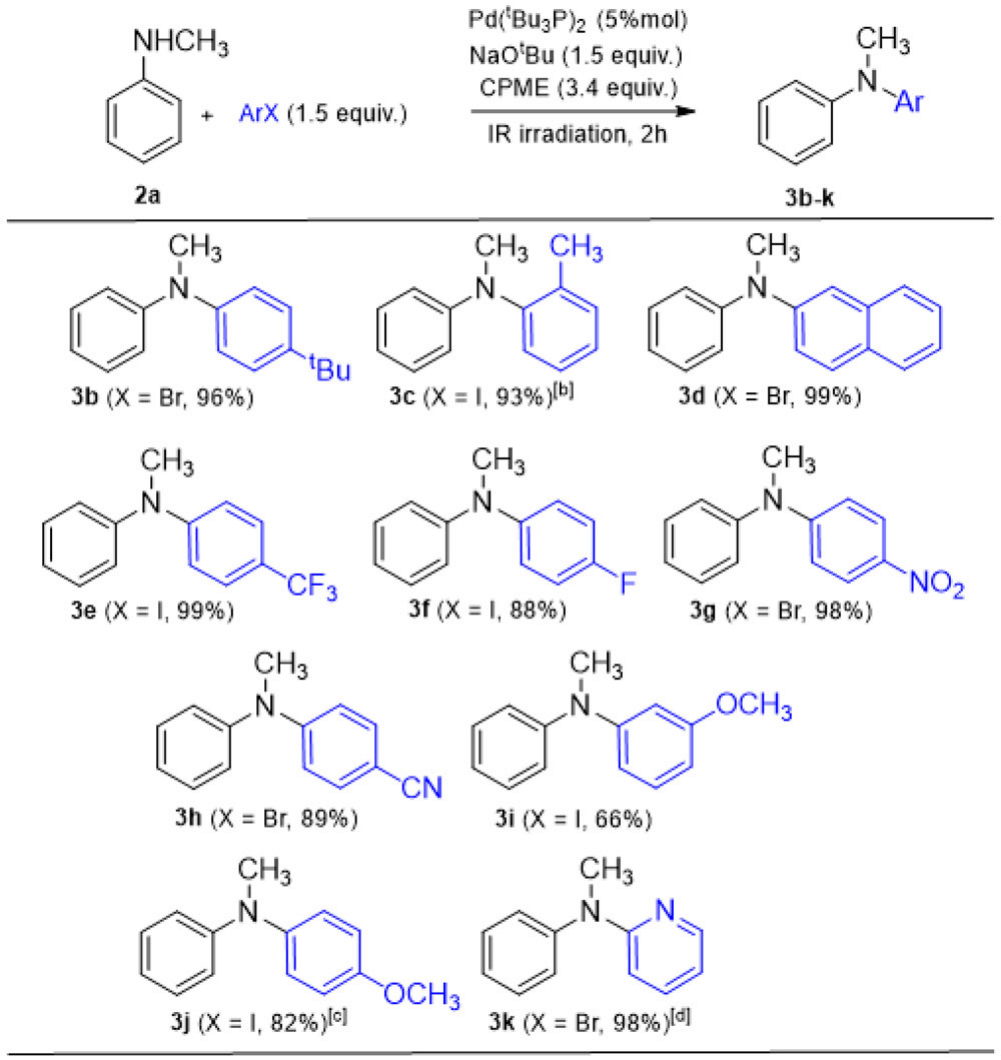
Aryl halide scope of the C–N cross‐coupling reactions.^[a].^ ^[a]^Reactions were carried out as follows: **2a** (1 mmol), ArX (1.5 mmol), Pd(^t^Bu_3_P)_2_ (5 mol%), NaO^t^Bu (1.5 mmol) in CPME (3.4 mmol) under IR irradiation for 2 hours. Unless specified otherwise, yields refer to isolated products (column chromatography). ^[b]^Reaction with 2‐bromo‐1,3‐dimethylbenzene afforded the coupling product in 11% isolated yield. ^[c]^Reaction with 1‐iodo‐2‐methoxybenzene afforded the coupling product in 42% yield (yield by ^1^H NMR analysis; it was not possible to separate the expected product from an impurity by chromatography). ^[d]^No formation of the expected product is observed using 4‐iodopyridine.

With the optimized conditions in hand (entries 9 and 12 of Table 1), we explored the scope of aryl halides by reacting *N*‐methylaniline **2a** with various aryl iodides and bromides. The results of this screening are summarized in Scheme 1. The Buchwald–Hartwig aminations proceeded in good to excellent yields with aryl halides bearing both electron‐donating groups, such as *tert*‐butyl (**3b**: 96%), methyl (**3c**: 93%), and methoxy (**3i**: 66% and **3j**: 82%), and electron‐withdrawing groups, including trifluoromethyl (**3e**: 99%), fluoro (**3f**: 88%), nitro (**3g**: 98%), and cyano (**3h**: 89%). Conversely, the reactions exhibited greater sensitivity to steric effects. While the coupling of **2a** with 2‐iodotoluene proceeded smoothly, yielding **3c** in 93%, the use of the more sterically hindered 2‐bromo‐1,3‐dimethylbenzene resulted in a significantly lower yield (11%, note [b] of Scheme 1). Similarly, the reaction of **2a** with 1‐iodo‐4‐methoxybenzene and 1‐iodo‐3‐methoxybenzene afforded the corresponding products in 82% and 66% yield, respectively, whereas the yield dropped to 42% when 1‐iodo‐2‐methoxybenzene was used (42%, note [c] of Scheme 1). Notably, the *N*‐arylated product was obtained in excellent yield with 2‐bromopyridine as the coupling partner (**3k**: 98%), whereas no formation of the expected product was observed when 4‐iodopyridine was used.

**Scheme 2:**
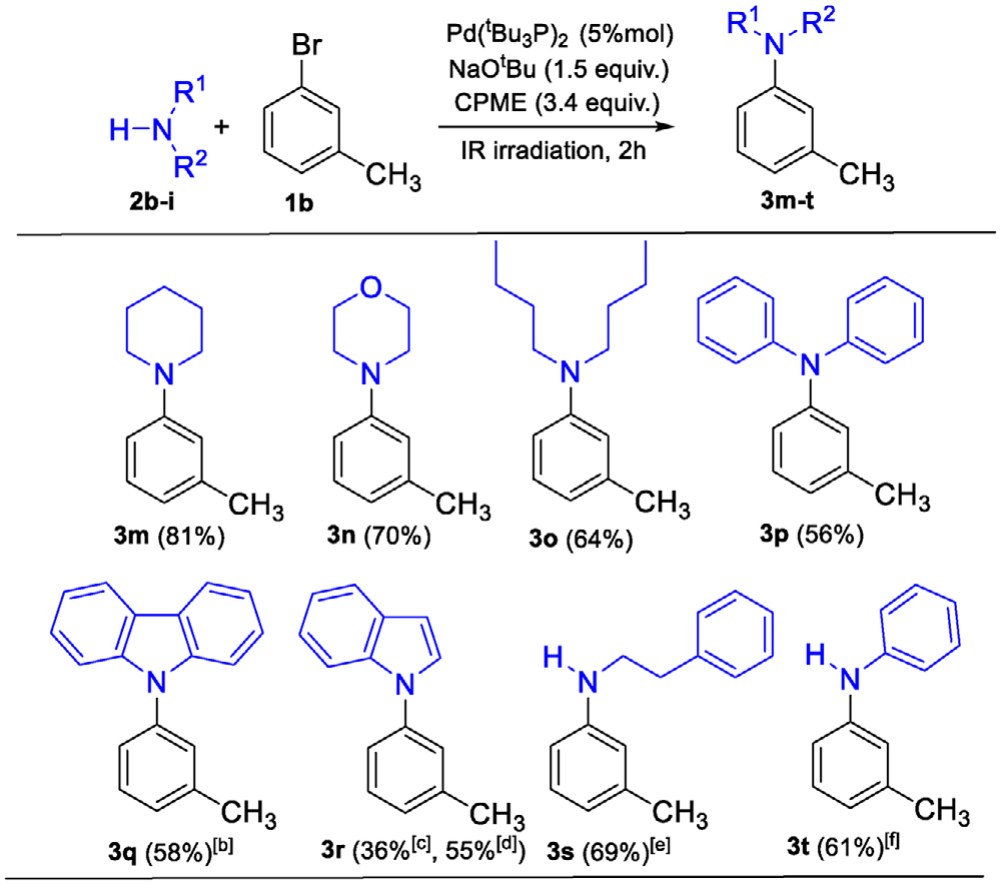
Amine scope of the C–N cross‐coupling reactions.^[a].^ ^[a]^Unless specified otherwise, reactions were carried out as follows: **2b‐i** (1 mmol), **1b** (1.5 mmol), Pd(^t^Bu_3_P)_2_ (5 mol%), NaO^t^Bu (1.5 mmol) in CPME (3.4 mmol) under IR irradiation for 2 hours. Yields refer to isolated products (column chromatography). ^[b]^The reaction was carried out using 6.8 equiv. of CPME. ^[c]^The formation of byproducts deriving from C‐arylation of the indole core was observed. ^[d]^ The reaction was carried out as follows: **1b** (1 mmol), 1*H*‐indole (1.5 mmol), Pd(^t^Bu_3_P)_2_ (5 mol%), NaO^t^Bu (1.5 mmol) in CPME (3.4 mmol) under IR irradiation for 2 hours. ^[e]^The reaction was carried out as follows: **1b** (1 mmol), 2‐phenylethylamine (6 mmol), Pd(^t^Bu_3_P)_2_ (5 mol%), NaO^t^Bu (1.5 mmol) in CPME (3.4 mmol) under IR irradiation for 2 hours (entry 6, Table 2). ^[f]^The reaction was carried out as follows: **1b** (1 mmol), aniline (3 mmol), Pd(^t^Bu_3_P)_2_ (5 mol%), NaO^t^Bu (1.5 mmol) in CPME (3.4 mmol) under IR irradiation for 2 hours (entry 2, Table 3); the reaction does not reach completion (87% conversion).

Next, to explore the scope of the amine substrate, various amines were reacted with 3‐bromotoluene **1b**, as shown in Scheme 2. Good yields were obtained with dialkylamines such as piperidine, morpholine, and dibutylamine (**3m**: 81%, **3n**: 70%, and **3o**: 64%, respectively). However, when diarylamines or heterocyclic amines, such as diphenylamine, 9*H*‐carbazole, and 1*H*‐indole, were used as coupling partners, the expected products were isolated in lower yields (**3p**: 56%, **3q**: 58%, and **3r**: 36%, respectively). Notably, in the reaction of **1b** with 1*H*‐indole, byproducts resulting from C‐arylation of the indole core were observed. Among the byproducts, a predominance of C/N *bis*‐arylated derivatives was detected. To improve the reaction outcome, the stoichiometry was adjusted by changing the **1b**:1*H*‐indole ratio from 1.5:1 to 1:1.5. This adjustment, using an excess of 1*H*‐indole, reduced the formation of C/N *bis*‐arylated byproducts and improved the reaction yield from 36% to 55%. The investigation was then extended to primary amines, such as 2‐phenylethylamine and aniline. Following the protocol outlined in entry 12 of Table 1, the coupling reactions of **1b** with 2‐phenylethylamine yielded a mixture of the expected *mono N*‐arylated product and the *bis N*‐arylated compound resulting from further arylation (Table 2). When a large excess of 2‐phenylethylamine (6 equiv.) was used, compound **3s** was isolated in 69% yield, representing the main product in the mixture reaction (88%). On the other hand, the reaction between **1b** and aniline predominantly produced the coupling product **3t**, with little to no other products (Table 3).

**Table 2:** Optimization of reaction conditions of **1b** with 2‐phenylethylamine.

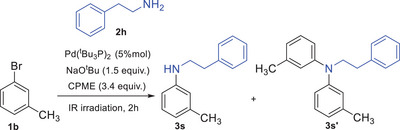		
Entry	1b:2h	3s:3s’
1	1.5:1	45:55
2	1:1	57:43
3	1:1.5	72:28
4	1:2	77:23
5	1:3	82:18
**6**	**1:6**	**88:12**

**Table 3:** Optimization of reaction conditions of **1b** with aniline.

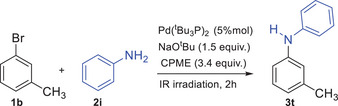		
Entry	1b:2i	Yield^[a]^
1^[b]^	1.5:1	50%
2^[c]^	1:3	61%

^[a]^ Yields refer to isolated products (column chromatography).

^[b]^ The reaction does not reach completion (90% conversion).

^[c]^ The reaction does not reach completion (87% conversion).

To evaluate the eco‐sustainability of our approach, we calculated the E‐factor (E), a widely accepted indicator that assesses the environmental impact of a chemical process by quantifying the waste generated per kilogram of the desired product.^[^ ^14^ ^]^ An ideal E‐factor is 0, with higher E‐factor values indicating greater waste generation. A key limitation of the E‐factor as a measure of greenness is that it does not account for the hazards of the generated waste, as it fails to differentiate between harmless and hazardous waste, and overlooks energy‐related waste. All the reactions in this study were carried out on a laboratory scale, requiring chromatography for accurate isolated yield measurements, a purification method known for its high waste production. To achieve a more realistic assessment of waste generation and, consequently, of the environmental impact of our synthetic approach, the synthesis of **3a** (Table 1, entry 9) was scaled up to a 10 mmol scale. The crude reaction mixture was then subjected to a short percolation on silica gel before being purified by distillation (Supporting information). Referring to this large‐scale reaction, where a purification method specifically suited to the reaction product was used instead of chromatography, we calculated both the complete E‐factor (cE) and the simple E‐factor (sE),^[^ ^14a^ ^]^ which were found to be 7.9 and 3.0, respectively (Supporting information).

## 3 Conclusion

We have reported here the first IR irradiation‐assisted Pd‐catalyzed Buchwald–Hartwig amination carried out under quasi‐solvent‐free conditions, in a nonanhydrous environment and without the exclusion of air. Our protocol is tolerant of various functional groups and can be applied to a range of secondary and primary amines, representing a novel, general, and green approach for C–N bond formation. The potential of our synthetic approach to mitigate environmental impact mainly arises from the use of i) CPME, an eco‐friendly solvent, as a reaction medium, ii) quasi‐solvent‐free conditions, iii) IR irradiation as an activating reaction mode. Its low toxicity makes CPME a suitable solvent to meet the principle 5 of Green Chemistry (safer solvents and auxiliaries), while its low peroxide formation rate, stability under both basic and acidic conditions, and relatively high boiling point minimize the potential for chemical accidents, aligning with principle 12 (accident prevention). Additionally, using a stoichiometric amount of solvent significantly also reduces reaction waste, in accordance with the principle 1 (waste prevention). On the other hand, NIR irradiation is a clean and efficient activating reaction mode, thanks to several key features of the emitter, including its immediate response time, high radiant efficiency, long lifetime of the tungsten filament, absence of emissions or pollution during operation, and cost‐effectiveness. As a result, NIR irradiation offers high efficiency with minimal environmental and economic impact, aligning with the principle 6 (design for energy efficiency). Finally, it is important to emphasize that performing reactions under nonanhydrous conditions and in the presence of air not only greatly simplifies the experimental procedures but also eliminates the need for auxiliary substances, such as inert gases, in line with the previously mentioned principle 5. Given these advantages, we believe our approach offers a greener synthetic alternative for amine synthesis and has the potential to inspire the development of new, sustainable protocols for palladium‐catalyzed cross‐coupling reactions. Further efforts will be needed to reduce waste generated during product isolation, thereby improving the overall eco‐sustainability of our protocol.

## Supporting Information

The authors have cited additional references within the Supporting Information.^[^ ^15^, ^16^, ^17^, ^18^, ^19^, ^20^, ^21^, ^22^, ^23^, ^24^, ^25^, ^26^, ^27^, ^28^ ^]^

## Conflict of Interests

The authors declare no conflict of interest.

## Supporting information



Supporting Information

## Data Availability

The data that support the findings of this study are available in the supplementary material of this article.
