# Supplementary material for: Infrared Irradiation‐Assisted Green Approach for Pd‐Catalyzed Buchwald–Hartwig Amination
Source: Chemistry. 2025 Apr 16;31(27):e202500557. doi: 10.1002/chem.202500557 (PMC12080305; doi:10.1002/chem.202500557)
Supplement: Supplementary file 1 — Supporting Information [file CHEM-31-e202500557-s001.pdf]

Supporting Information

Table of contents

Synthetic Procedures S2

^{1}H-NMR, ^{19}F-NMR and ^{13}C-NMR spectra S8

Calculation of E-factor values..S29

Supplementary References...S30

Synthetic Procedures

General remarks: All reagents and solvents were purchased at the highest commercial purity and used without further purification. Preparative column chromatography was carried out using Macherey-Nagel silica gel (60, particle size 0.040-0.063 mm). Macherey-Nagel aluminium sheets with silica gel 60 F254 were used for TLC analyses. GC analyses were performed on an Agilent 7890B equipped with a Agilent J&W 5MS capillary column (30 m×0.25 mm id). GC-MS analyses were performed on a Thermo Polaris Q spectrometer equipped with a Macherey-Nagel Optima-1 capillary column (30 m×0.25 mm id), ionization mode EI (70 eV). ^{1}H NMR and ^{13}C NMR spectra were acquired on an Agilent 500 spectrometer at 500 and at 126 MHz, respectively, using the $CDCl_3$ residual proton peak at δ =7.26 ppm as internal standard for ^{1}H spectra and the signals of $CDCl_3$ at δ = 77.16 ppm as internal standard for ^{13}C spectra. ^{19} F NMR spectra were acquired on an Agilent 500 spectrometer at 470 MHz. IR irradiation-assisted reactions were carried out using a 250W infrared incandescent lamp (Philips, 230-250 V BR125). These lamps have a broad emission spectrum, but most of the radiation they produce is in the near-infrared (NIR) region (0.76-2 μm) with maximum intensity at about 1.2 μm. The distance between the lamp bulb and the reactor, a Carius tube mounted on the side of a IR lamp, was set to 4 cm as in Figure S1 (the temperature reached in the Carius tube was about 110 °C). To measure the internal temperature, a Carius tube equipped with a perforated screw cap and a tight gasket, allowing the insertion of a thermometer into the reaction medium, was used.

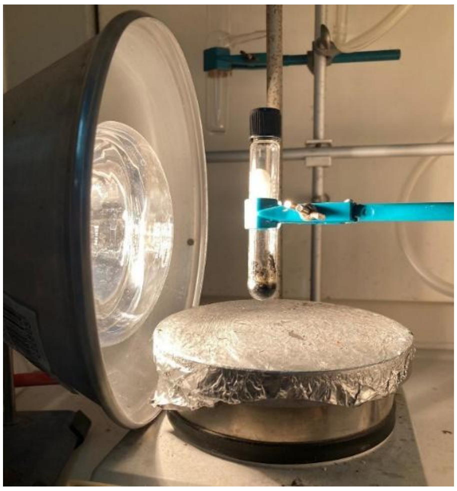

Figure S1. Experimental set-up.

General procedure. A Carius tube (ø=1.5 cm) with a screw cap and equipped with a magnetic stirrer was charged with sodium *tert*-butoxide (1.5 equiv), $Pd(^{t}Bu_3P)_2$ (5 mol %) and CMPE (3.4 equiv), then amine (1 equiv) and aryl halide (1.5 equiv) were added. The reaction mixture was reacted under IR irradiation and magnetic stirring. After 2h, the mixture was cooled to room temperature, diluted with water and extracted with ethyl acetate. The organic extracts were washed with an aqueous solution of brine, dried with Na_2SO_4 and concentrated under vacuum.

The crude product was purified by column chromatography on silica gel. The acquired ^{1}H and ^{13}C spectra of all the synthesized compounds were compared with literature data and found to be in agreement.

N,3-dimethyl-N-phenylaniline ***3a***.[15] Compound **3a** was synthesized from *N*-methylaniline **2a** (108 µl, 1 mmol) and 3-bromotoluene (182 µl, 1.5 mmol) in accordance with the general procedure. The crude product was purified by column chromatography (hexane) afforded 189 mg of compound **3a** (96% yield). MS (EI, 70 eV) *m/z* (%): 197 (100) $[M]^{+\bullet}$; ^{1}H NMR (500 MHz, $CDCl_3$) δ = 7.29 (dd like, *J* = 8.6, 7.3 Hz, 2H), 7.19 (t, *J* = 7.7 Hz, 1H), 7.03 (dd like, *J* = 8.7, 1.0 Hz, 2H), 6.96 (tt, *J* = 7.4, 1.0 Hz, 1H), 6.89-6.85 (m, 2H), 6.83-6.80 (m, 1H), 3.33 (s, 3H), 2.33 (s, 3H); ^{13}C NMR (126 MHz, $CDCl_3$) δ = 149.3, 149.2, 139.1, 129.3, 129.2, 122.5, 121.6, 121.1, 120.2, 118.1, 40.0, 21.7.

4-(tert-butyl)-N-methyl-N-phenylaniline ***3b***.[16]

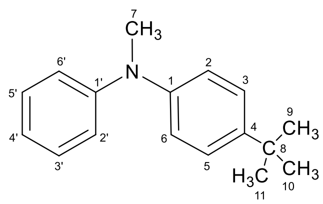

Compound **3b** was synthesized from *N*-methylaniline **2a** (108 µl, 1 mmol) and 1-bromo-4-(*tert*-butyl)benzene (260 µl, 1.5 mmol) in accordance with the general procedure. The crude product was purified by column chromatography (hexane) affording 230 mg of compound **3b** (96% yield). MS (EI, 70 eV) *m/z* (%): 239 (63) $[M]^{+\bullet}$, 224 (100) $[M - CH_3]^{+}$; ^{1}H NMR (500 MHz, $CDCl_3$) δ = 7.32 (d like, *J* = 8.8 Hz, 2H, $H_{3,5}$), 7.26 (dd like, *J* = 8.7, 7.3 Hz, 2H, $H_{3',5'}$), 7.02 (d like, *J* = 8.8 Hz, 2H, $H_{2,6}$), 6.98 (dd like, *J* = 8.7, 1.0 Hz, 2H, $H_{2',6'}$), 6.91 (tt, *J* = 7.4, 1.1 Hz, 1H, $H_{4'}$), 3.31 (s, 3H, H_7), 1.33 (s, 9H, $H_{9,10,11}$); ^{13}C NMR (126 MHz, $CDCl_3$) δ = 149.3 (C_{Ar}), 146.5 (C_{Ar}), 144.9 (C_{Ar}), 129.2 (C_{Ar}), 126.2 (C_{Ar}), 121.4 (C_{Ar}), 120.4 (C_{Ar}), 119.1(C_{Ar}) , 40.4 (C_7), 34.4 (C_8), 31.6 ($C_{9,10,11}$).

N,2-dimethyl-N-phenylaniline ***3c***.[17] Compound **3c** was synthesized from *N*-methylaniline **2a** (108 µl, 1 mmol) and 2-iodotoluene (192 µl, 1.5 mmol) in accordance with the general procedure. The crude product was purified by column chromatography (hexane) affording 183 mg of compound **3c** (93% yield). MS (EI, 70 eV) *m/z* (%): 197 (100) $[M]^{+\bullet}$, 182 (37); ^{1}H NMR (500 MHz, $CDCl_3$) δ = 7.32-7.29 (m, 1H), 7.28-7.23 (m, 1H), 7.23-7.14 (m, 4H), 6.72 (tt, *J* = 7.3, 1.0 Hz, 1H), 6.55 (dd like, *J* = 8.8, 1.0 Hz, 2H), 3.24 (s, 3H), 2.16 (s, 3H); ^{13}C NMR (126 MHz, $CDCl_3$) δ = 149.3, 146.9, 137.0, 131.5, 129.1, 128.5, 127.6, 126.5, 116.9, 113.0, 39.2, 18.0.

N-methyl-N-phenylnaphthalen-2-amine ***3d***.[15] Compound **3d** was synthesized from *N*-methylaniline **2a** (108 µl, 1 mmol) and 2-bromonapthalene (311 mg, 1.5 mmol) in accordance with the general procedure. The crude product was purified by column chromatography (from hexane to hexane:ethyl acetate 9.5:0.5) affording 231 mg of compound **3d** (99% yield). MS (EI, 70 eV) *m/z* (%): 233 (100) $[M]^{+\bullet}$, 232 (61), 217 (54); ^{1}H NMR (500 MHz, $CDCl_3$) δ 7.74 (d, *J* = 8.0 Hz, 1H), 7.69 (t, *J* = 8.4 Hz, 2H), 7.42 (t, *J* = 7.4 Hz, 1H), 7.35-7.29 (m, 4H), 7.22 (br d, *J* = 8.9 Hz, 1H),

7.11 (d, *J* = 7.9 Hz, 2H), 7.02 (t, *J* = 7.1 Hz, 1H), 3.43 (s, 3H); ^{13}C NMR (126 MHz, $CDCl_3$) δ = 149.2, 146.7, 134.8, 129.4, 129.2, 128.7, 127.7, 126.9, 126.4, 123.8, 122.1, 121.9, 121.5, 114.7, 40.8.

N-methyl-N-phenyl-4-(trifluoromethyl)aniline ***3e***.[16] Compound **3e** was synthesized from *N*-methylaniline **2a** (108 μl, 1 mmol) and 1-iodo-4-(trifluoromethyl)benzene (220 μl, 1.5 mmol) in accordance with the general procedure. The crude product was purified by column chromatography (from hexane to hexane:ethyl acetate 9.5:0.5) affording 249 mg of compound **3e** (99% yield). MS (EI, 70 eV) *m/z* (%): 251(100) $[M]^{+\bullet}$, 250 (67); ^{1}H NMR (500 MHz, $CDCl_3$) δ = 7.42 (d like, *J* = 8.9 Hz, 2H), 7.39 (t like, *J* = 7.9 Hz, 2H), 7.21-7.16 (m, 3H), 6.85 (d like, *J* = 8.9 Hz, 2H), 3.35 (s, 3H); ^{19}F NMR (470 MHz MHz, $CDCl_3$) δ = -61.25;^{13}C NMR (126 MHz, $CDCl_3$) δ = 151.6, 148.0, 129.9, 126.3 (q, *J* = 3.8 HZ), 125.4, 125.1, 125.0 (q, *J* = 270 Hz), 120.1 (q, *J* = 32.6 Hz), 115.0, 40.3.

4-Fluoro-N-methyl-N-phenylaniline ***3f***.[16] Compound **3f** was synthesized from *N*-methylaniline **2a** (108 μl, 1 mmol) and 1-fluoro-4-iodobenzene (173 μl, 1.5 mmol) in accordance with the general procedure. The crude product was purified by column chromatography (hexane) affording 177 mg of compound **3f** (88% yield). MS (EI, 70 eV) *m/z* (%): 201 (100) $[M]^{+\bullet}$, 200 (67), 185 (39), 122 (23); ^{1}H NMR (500 MHz, $CDCl_3$) δ = 7.27-7.22 (m, 2H), 7.07-6.97 (m, 4H), 6.92-6.87 (m, 3H). 3.28 (s, 3H); ^{19}F NMR (470 MHz, $CDCl_3$) δ = - 120.7;^{13}C NMR (126 MHz, $CDCl_3$) δ = 158.8 (d, *J* = 242 Hz), 149.4, 145.4 (d, *J* = 2.7 Hz), 129.3, 124.4 (d, *J* = 7.9 Hz), 120.2, 118.2, 116.1 (d, *J* = 22.3 Hz), 40.7.

N-Methyl-4-nitro-N-phenylaniline ***3g***.[18] Compound **3g** was synthesized from *N*-methylaniline **2a** (108 μl, 1 mmol) and 1-bromo-4-nitrobenzene (303 mg, 1.5 mmol) in accordance with the general procedure. The crude product was purified by column chromatography (hexane:ethyl acetate 8.5:1.5) affording 224 mg of compound **3g** (98% yield). ^{1}H NMR (500 MHz, $CDCl_3$) δ = 8.06 (d like, *J* = 9.4 Hz, 2H), 7.48-7.43 (m, 2H), 7.31 (t like, *J* = 7.5 Hz, 1H), 7.25-7.21 (m, 2H), 6.67 (d like, *J* = 9.4 Hz, 2H), 3.41 (s, 3H); ^{13}C NMR (126 MHz, $CDCl_3$) δ = 153.9, 146.6, 138.4, 130.4, 126.9, 126.8, 125.9, 112.6, 40.7.

4-(Methyl(phenyl)amino)benzonitrile ***3h***.[19] Compound **3h** was synthesized from *N*-methylaniline **2a** (108 μl, 1 mmol) and 4-bromobenzonitrile (273 mg, 1.5 mmol) in accordance with the general procedure. The crude product was purified by column chromatography (hexane:ethyl acetate 8:2) affording 185 mg of compound **3h** (89% yield). MS (EI, 70 eV) *m/z* (%): 208 (100) $[M]^{+\bullet}$, 207 (83), 192 (31), 129 (18); ^{1}H NMR (500 MHz, $CDCl_3$) δ = 7.48-7.42 (m, 4H), 7.28 (t, *J* = 7.2 Hz, 1H), 7.22 (d, *J* = 7.8 Hz, 2H), 6.75 (d, *J* = 8.7 Hz, 2H), 3.37 (s, 3H) ; ^{13}C NMR (126 MHz, $CDCl_3$) δ = 152.0, 146.9, 133.3, 130.2, 126.6, 126.3, 120.5, 113.9, 99.4, 40.3.

3-Methoxy-N-methyl-N-phenylaniline ***3i***.[16] Compound **3i** was synthesized from *N*-methylaniline **2a** (108 μl, 1 mmol) and 1-iodo-3-methoxybenzene (178 μl, 1.5 mmol) in accordance with the general procedure. The crude product was purified by column chromatography (from hexane to hexane:ethyl acetate 9.5:0.5) affording 141 mg of compound **3i** (66% yield). MS (EI, 70 eV) *m/z* (%): 213 (100) $[M]^{+\bullet}$, 212 (52), 197 (18); ^{1}H NMR (500 MHz,

$CDCl_3$) δ = 7.32-7.27 (m, 2H), 7.16 (t, *J* = 8.1 Hz, 1H), 7.09-7.05 (m, 2H), 7.02-6.97 (m, 1H), 6.58 (ddd, *J* = 8.1, 2.1, 0.7 Hz, 1H), 6.54 (t, *J* = 2.3 Hz, 1H), 6.49 (ddd, *J* = 8.1, 2.4, 0.6 Hz, 1H), 3.76 (s, 3H), 3.31 (s, 3H); ^{13}C NMR (126 MHz, $CDCl_3$) δ = 160.6, 150.5, 149.0, 129.9, 129.4, 122.1, 121.8, 112.3, 105.9, 105.6, 55.3, 40.4.

4-Methoxy-N-methyl-N-phenylaniline ***3j***.[20] Compound **3j** was synthesized from *N*-methylaniline **2a** (108 ml, 1 mmol) and 1-iodo-4-methoxybenzene (351 mg, 1.5 mmol) in accordance with the general procedure. The crude product was purified by column chromatography (hexane:ethyl acetate 9.9:0.1) affording 175 mg of compound **3j** (82% yield). MS (EI, 70 eV) *m/z* (%): 213 (81) $[M]^{+•}$, 199 (16), 198 (100); ^1H NMR (500 MHz, $CDCl_3$) δ = 7.23-7.18 (m, 2H), 7.12-7.08 (m, 2H), 6.92-.6.88 (m, 2H), 6.82-6.77 (m, 3H), 3.82 (s, 3H), 3.26 (s, 3H); ^{13}C NMR (126 MHz, $CDCl_3$) δ = 156.4, 149.8, 142.3, 129.1, 126.3, 118.5, 115.9, 114.9, 55.7, 40.7.

N-methyl-N-phenylpyridin-2-amine ***3k***.[17] Compound **3k** was synthesized from *N*-methylaniline **2a** (108 ml, 1 mmol) and 2-bromopyridine (143 μl, 1.5 mmol) in accordance with the general procedure. The crude product was purified by column chromatography (hexane:ethyl acetate 8:2) affording 180 mg of compound **3k** (98% yield). MS (EI, 70 eV) *m/z* (%): 184 (24) $[M]^{+•}$, 183 (100), 168 (15); ^1H NMR (500 MHz, $CDCl_3$) δ = 8.23 (ddd, *J* = 5.0, 1.9, 09 Hz, 1H), 7.42-7.37 (m, 2H), 7.30 (ddd, *J* = 9.0, 7.1, 2.0 Hz, 1H), 7.28-7.25 (m 2H), 7.23-7.19 (m, 1H), 6.60 (ddd, *J* = 7.0, 5.0, 0.9 Hz, 1H), 6.53 (dt, *J* = 8.6, 0.9 Hz, 1H), 3.48 (s, 3H); ^{13}C NMR (126 MHz, $CDCl_3$) δ = 159.0, 147.9, 147.0, 136.7, 129.8, 126.5. 125.6, 113.2, 109.3, 38.5.

1-(m-Tolyl)piperidine ***3m***.[21] Compound **3m** was synthesized from piperidine (99 μl, 1 mmol) and 3-bromotoluene (182 μl, 1.5 mmol) in accordance with the general procedure. The crude product was purified by column chromatography (hexane:ethyl acetate 9.9:0.1) affording 142 mg of compound **3m** (81% yield). MS (EI, 70 eV) *m/z* (%): 175 (71) $[M]^{+•}$, 174 (100), 146 (18), 134 (10), 119 (14), 118 (29), 91 (17); ^1H NMR (500 MHz, $CDCl_3$) δ = 7.15 (t, *J* = 7.7 Hz, 1H), 6.80-6.75 (m, 2H), 6.67 (br d, *J* = 7.5 Hz, 1H), 3.15 (t, *J* = 5.5 Hz, 4H), 2.33 (s, 3H), 1.72 (quintet, J = 5.6 Hz, 4H), 1.61-1.55 (m, 2H); ^{13}C NMR (126 MHz, $CDCl_3$) δ = 152.5, 138.7, 129.0, 120.3, 117.6, 113.8, 50.9, 26.1, 24.5, 21.9.

4-(m-Tolyl)morpholine ***3n***.[21] Compound **3n** was synthesized from morpholine (87 μl, 1 mmol) and 3-bromotoluene (182 μl, 1.5 mmol) in accordance with the general procedure. The crude product was purified by column chromatography (hexane:ethyl acetate 9.5:0.5) affording 124 mg of compound **3n** (70% yield). MS (EI, 70 eV) *m/z* (%): 177 (100) $[M]^{+•}$, 176 (13), 146 (11), 132 (13), 119 (80), 118 (74), 91 (47); ^1H NMR (500 MHz, $CDCl_3$) δ = 7.17 (t, *J* = 7.7 Hz, 1H), 6.75-6.70 (m, 3H), 3.87-3,84 (m, 4H), 3.17-3.13 (m, 4H), 2.33 (s, 3H); ^{13}C NMR (126 MHz, $CDCl_3$) δ = 151.5, 139.0, 129.2, 121.1, 116.7, 113.0, 67.1, 49.6, 21.9.

N,N-Dibutyl-3-methylaniline ***3o***.[22]

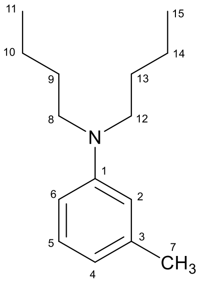

Compound **3o** was synthesized from dibutylamine (169 μl, 1 mmol) and 3-bromotoluene (182 μl, 1.5 mmol) in accordance with the general procedure. The crude product was purified by column chromatography (from hexane to hexane:CH_2Cl_2 9.5:0.5) affording 140 mg of compound **30** (64% yield). MS (EI, 70 eV) *m/z* (%): 219 (22) $[M]^{+\bullet}$, 176 (46) $[M - CH_3CH_2CH_2]^+$, 134 (100) $[CH_2{=}N(CH_3)Ar]^+$, 120 (14) $[CH_2{=}NHAr]^+$; ^{1}H NMR (500 MHz, $CDCl_3$) δ = 7.11 (dd like, *J* = 9.2, 6.9 Hz, 1H, H_5), 6.51-6.46 (m, 3H, $H_{2,4,6}$), 3.27 (t, *J* = 7.6 Hz, 4H, $H_{8,12}$), 2.33 (s, 3H, H_7), 1.62-1.54 (m, 4H, $H_{9,13}$), 1.42-1.33 (m, 4H, $H_{10,14}$), 0.98 (t, *J* = 7.4 Hz, 6H, $H_{11,15}$); ^{13}C NMR (126 MHz, $CDCl_3$) δ = 148.4 (C_{Ar}), 138.9 (C_{Ar}), 129.2 (C_{Ar}), 116.2 (C_{Ar}), 112.5 (C_{Ar}), 109.1 (C_{Ar}), 50.9 ($C_{8,12}$), 29.6 ($C_{9,13}$), 22.2 (C_7), 20.5 ($C_{10,14}$), 14.2 ($C_{11,15}$).

3-Methyl-N,N-diphenylaniline ***3p***.[23] Compound **3p** was synthesized from diphenylamine (169 mg, 1 mmol) and 3-bromotoluene (182 μl, 1.5 mmol) in accordance with the general procedure. The crude product was purified by column chromatography (hexane:ethyl acetate 9.9:0.1) affording 146 mg of compound **3p** (53% yield). MS (EI, 70 eV) *m/z* (%): 259 (100) $[M]^{+\bullet}$, 258 (31), 244 (17), 243 (19); ^{1}H NMR (500 MHz, $CDCl_3$) δ = 7.26-7.21 (m, 4H), 7.14 (t, *J* = 7.8 Hz, 1H), 7.10-7.06 (m, 4H), 7.02-6.98 (m, 2H), 6.92 (br s, 1H), 6.91-6.87 (m, 1H), 6.84 (d, *J* = 7.5 Hz, 1H), 2.26 (s, 3H); ^{13}C NMR (126 MHz, $CDCl_3$) δ = 148.1, 147.9, 139.2, 129.3, 129.2, 125.1, 124.2, 123.8, 122.6, 121.7, 21.6.

9-(m-Tolyl)-9H-carbazole ***3q***.[24] Compound **3q** was synthesized from carbazole (167 mg, 1 mmol) and 3-bromotoluene (182 μl, 1.5 mmol) in accordance with the general procedure (6.8 equiv. of CPME were used instead of 3.4 equiv.). The crude product was purified by column chromatography (hexane) affording 150 mg of compound **3p** (58% yield). MS (EI, 70 eV) *m/z* (%) : 257 (100) $[M]^{+\bullet}$, 256 (14), 242 (12), 241 (21), 127 (13); ^{1}H NMR (500 MHz, $CDCl_3$) δ = 8.19 (dd, *J* = 7.7, 0.7 Hz, 2H), 7.52 (t, *J* = 7.6 Hz, 1H), 7.47-7.38 (m, 6H), 7.34-7.29 (m, 3H), 2.50 (s, 3H); ^{13}C NMR (126 MHz, $CDCl_3$) δ = 141.1, 140.0, 137.7, 129.7, 128.4, 127.8, 126.0, 124.3, 123.4, 120.4, 119.9, 110.0, 21.6.

1-(m-Tolyl)-1H-indole ***3r***.[24] Compound **3r** was synthesized from 3-bromotoluene (121 μl, 1 mmol), 1*H*-indole (176 mg, 1.5 mmol), Pd(^{t}Bu$_3$P)$_2$ (26 mg, 5 mol%), NaO^{t}Bu (144 mg, 1.5 mmol) in CPME (0.4 mL, 3.4 mmol) under IR irradiation for 2 h. The crude product was purified by column chromatography (hexane) affording 114 mg of compound **3r** (55% yield). MS (EI, 70 eV) *m/z* (%): 207 (100) $[M]^{+\bullet}$, 206 (27), 192 (11), 89 (15); ^{1}H NMR (500 MHz, $CDCl_3$) δ = 7.74-7.71 (m, 1H), 7.61 (dd, *J* = 8.2, 0.8 Hz, 1H), 7.45-7.40 (m, 1H), 7.38-7.33 (m, 3H), 7.28-7.23 (m, 1H), 7,23-

7.18 (m, 2H), 6.71 (dd, *J* = 3.2, 0.8 Hz, 1H), 2.48 (s, 3H); ^{13}C NMR (126 MHz, $CDCl_3$) δ = 139.9, 139.8, 136.0, 129.5, 129.4, 128.1, 127.3, 125.1, 122.4, 121.5, 121.2, 120.4, 110.7, 103.5, 21.6.

*3-Methyl-N-phenethylaniline **3s***.[26] Compound **3s** was synthesized from 3-bromotoluene (121 μl, 1 mmol) and 2-phenylethylamine (0.76 mL, 6 mmol) under the synthetic protocol defined in the entry 6 of **Table 2**. The crude product was percoled on silica gel (hexane:ethyl acetate 9.9:0.1) to remove the large excess of 2-phenylethylamine, then purified by column chromatography (silica gel pretreated with a mixture of hexane:ethyl acetate 9.5:0.5 containing 1% of Et_3N, hexane:ethyl acetate 9.5:0.5) affording 146 mg of compound **3s** (69% yield). MS (EI, 70 eV) *m/z* (%): 211 (13) $[M]^{+\bullet}$, 120 (100), 91 (24); ^{1}H NMR (500 MHz, $CDCl_3$) δ = 7.36-7.31 (m, 2H), 7.27-7.23 (m, 3H), 7.11-7.06 (m, 1H), 6.57-6.54 (m, 1H), 6.47-6.43 (m, 2H), 3.64 (br s, 1H), 3.41 (t, *J* = 7.0 Hz, 2H), 2.93 (t, *J* = 7.0 Hz, 2H), 2.29 (s, 3H); ^{13}C NMR (126 MHz, $CDCl_3$) δ = 148.2, 139.5, 139.2, 129.3, 128.9, 128.7, 126.5, 118.5, 113.9, 110.3, 45.2, 35.7, 21.8.

*3-Methyl-N-phenylaniline **3t***.[27] Compound **3t** was synthesized from 3-bromotoluene (121 ml, 1 mmol) and aniline (0.27 mL, 3 mmol) under the synthetic protocol defined in the entry 2 of **Table 3**. The crude product was purified by column chromatography (silica gel pretreated with a mixture of hexane:ethyl acetate 9.5:0.5 containing 1% of Et_3N, hexane:ethyl acetate 9.5:0.5) affording 112 mg of compound **3t** (61% yield). MS (EI, 70 eV) *m/z* (%): 183 (100) $[M]^{+\bullet}$, 182 (47), 168 (24), 167 (43); ^{1}H NMR (500 MHz, $CDCl_3$) δ = 7.30-7.25 (m, 2H), 7.16 (td, *J* = 7.4, 1.2 Hz, 1H), 7.08 (dd, *J* = 8.5, 1.0 Hz, 2H), 6.96-6.88 (m, 3H), 6.77 (br d, J = 7.6 Hz, 1H), 5.75 (br s, 1H), 2.32 (s, 3H); ^{13}C NMR (126 MHz, $CDCl_3$) δ = 143.3, 143.1, 139.4, 129.5, 129.3, 122.1, 121.0, 118.6, 118.0, 115.1, 21.7.

^{1}H-NMR, ^{19}F-NMR and ^{13}C-NMR spectra

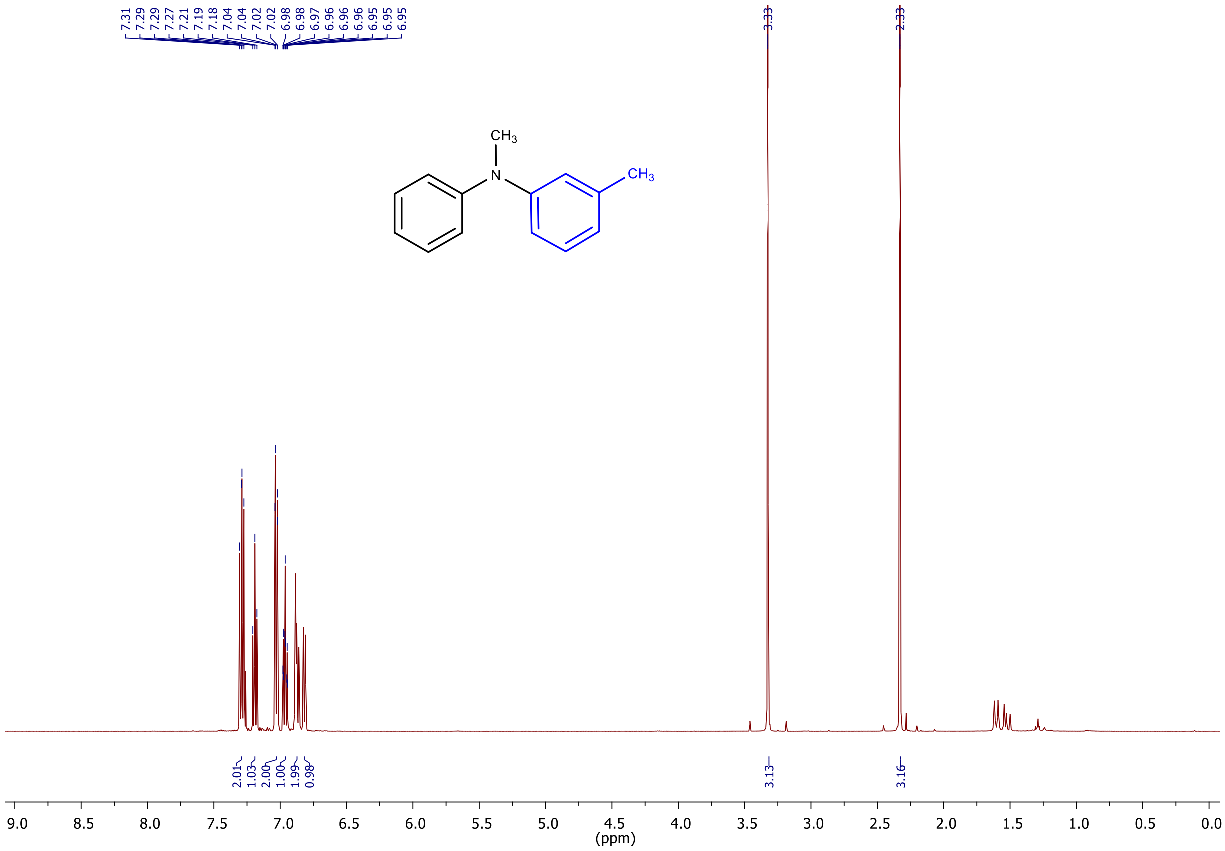

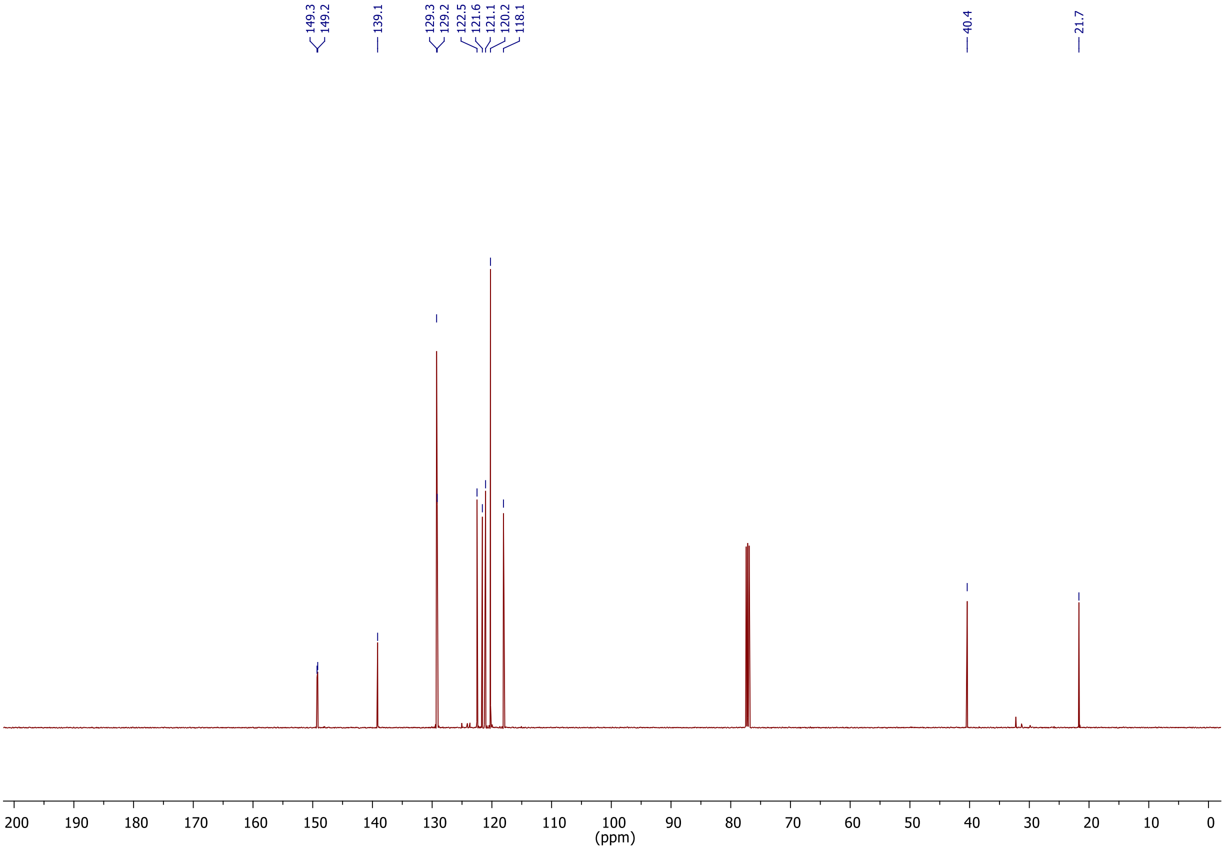

Figure S2. ^{1}H NMR and ^{13}C NMR spectra of compound **3a** (500 and 126 MHz, $CDCl_3$).

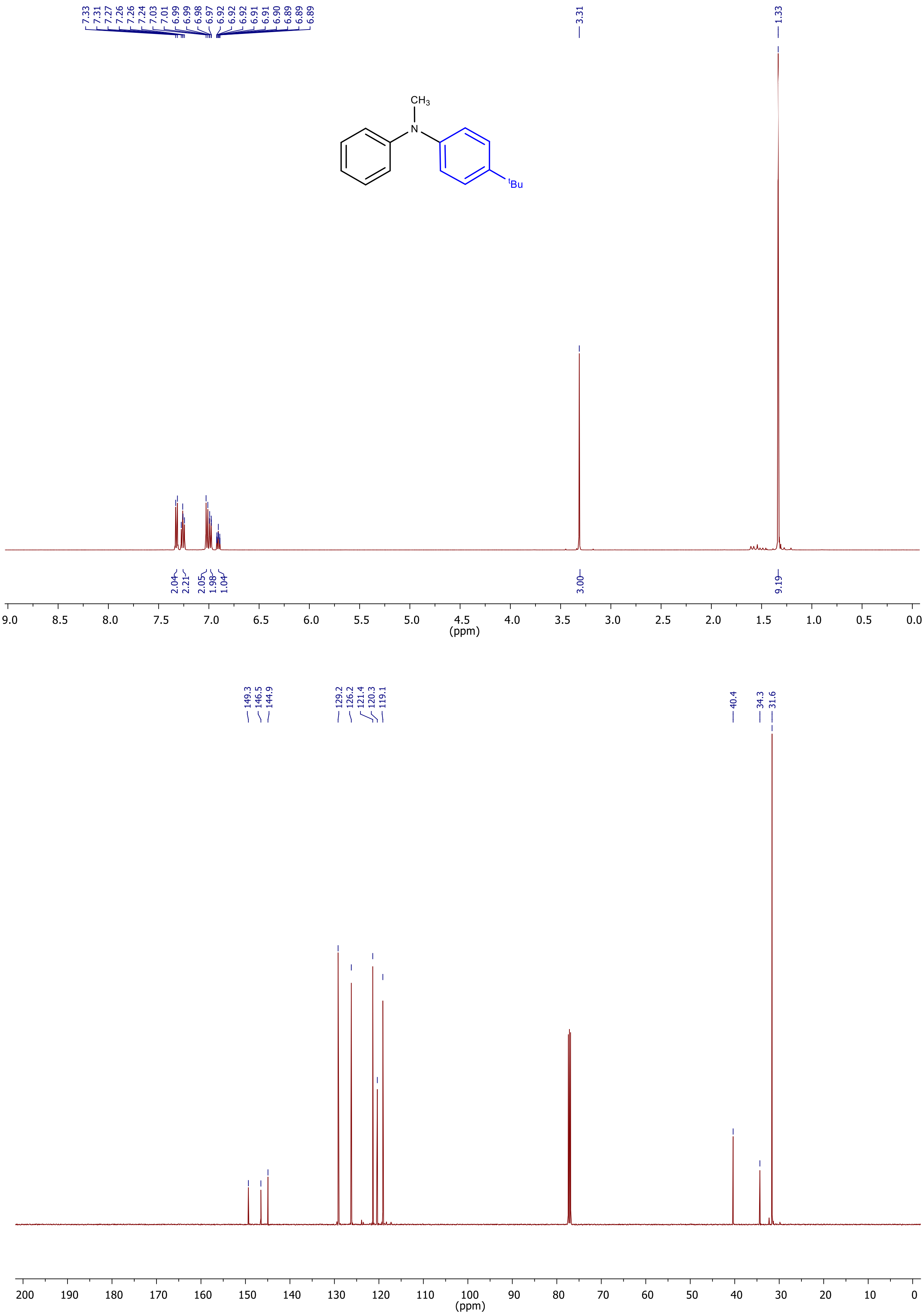

Figure S3. ^{1}H NMR and ^{13}C NMR spectra of compound **3b** (500 and 126 MHz, $CDCl_3$).

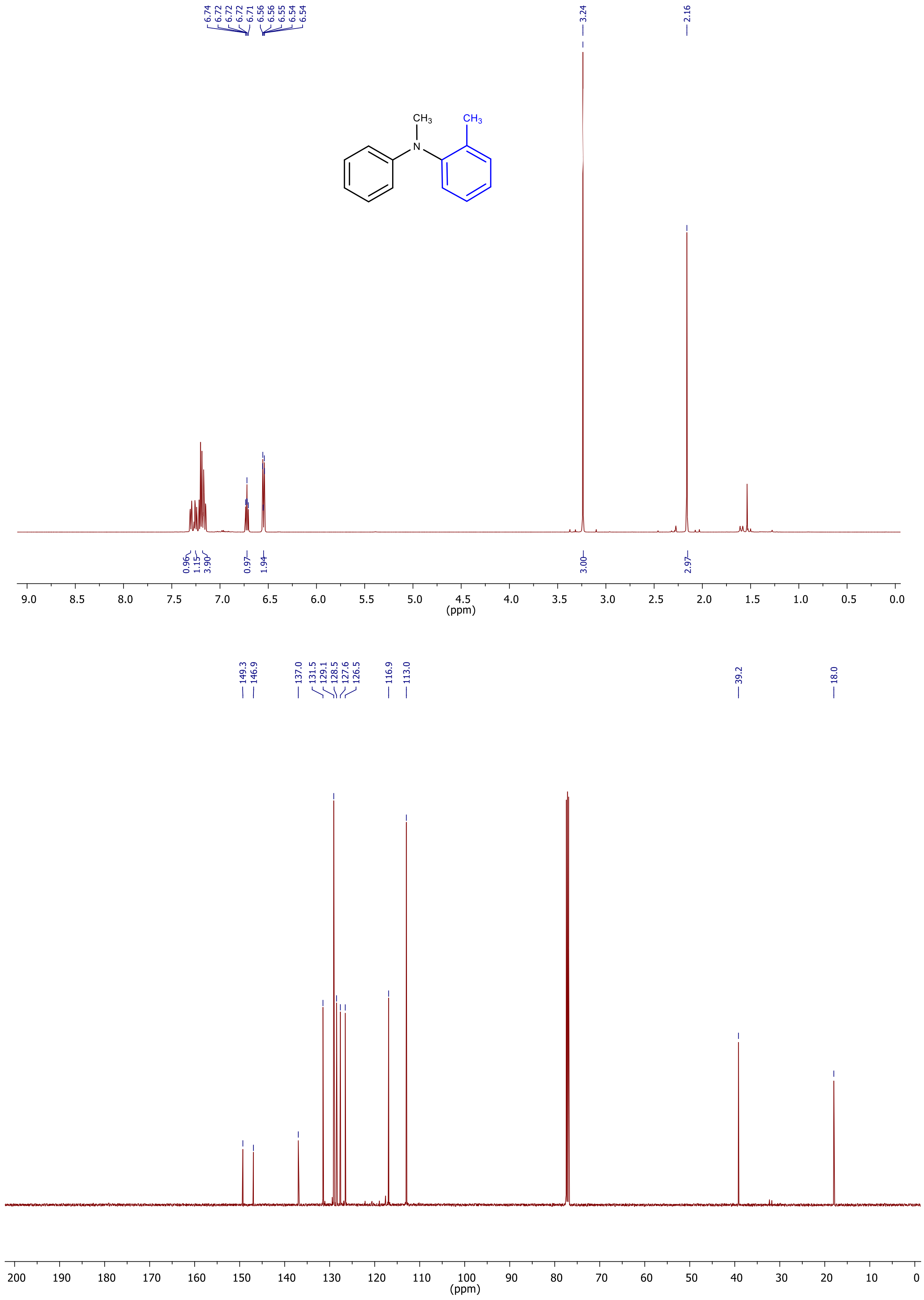

Figure S4. ^{1}H NMR and ^{13}C NMR spectra of compound **3c** (500 and 126 MHz, $CDCl_3$).

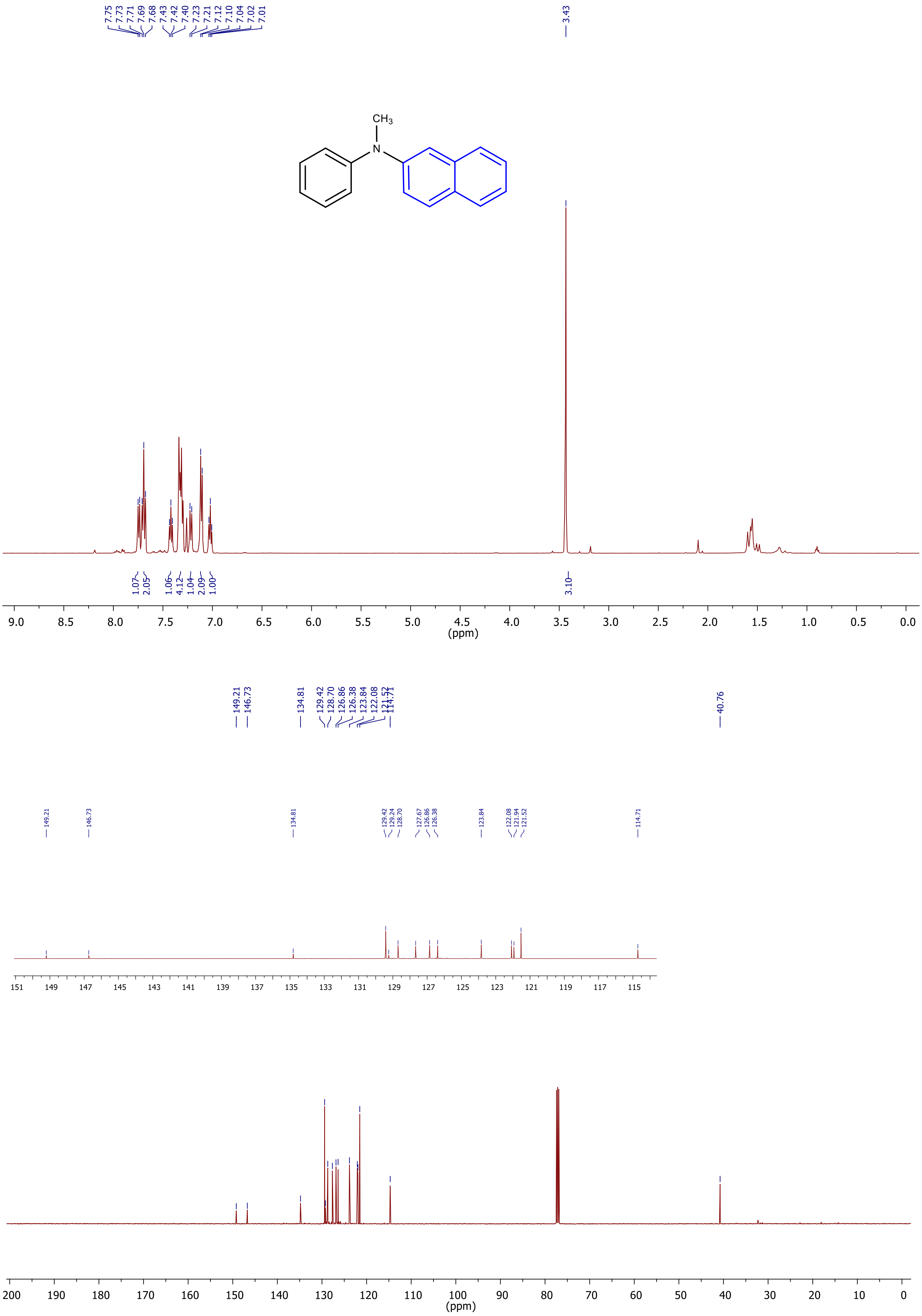

Figure S5. ^{1}H NMR and ^{13}C NMR spectra of compound **3d** (500 and 126 MHz, $CDCl_3$).

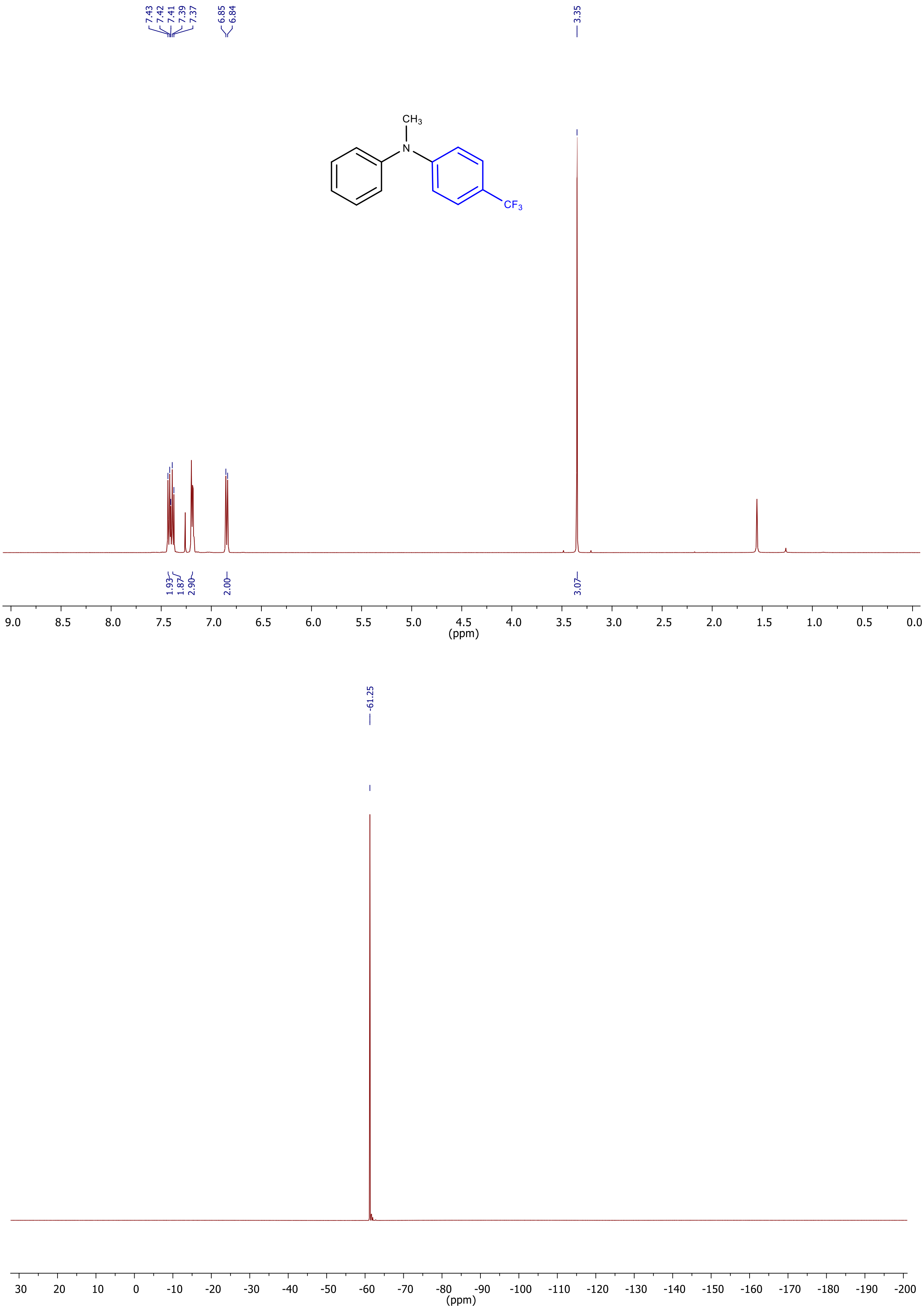

Figure S6a. ^{1}H NMR and ^{19}F NMR spectra of compound **3e** (500 and 470 MHz, $CDCl_3$).

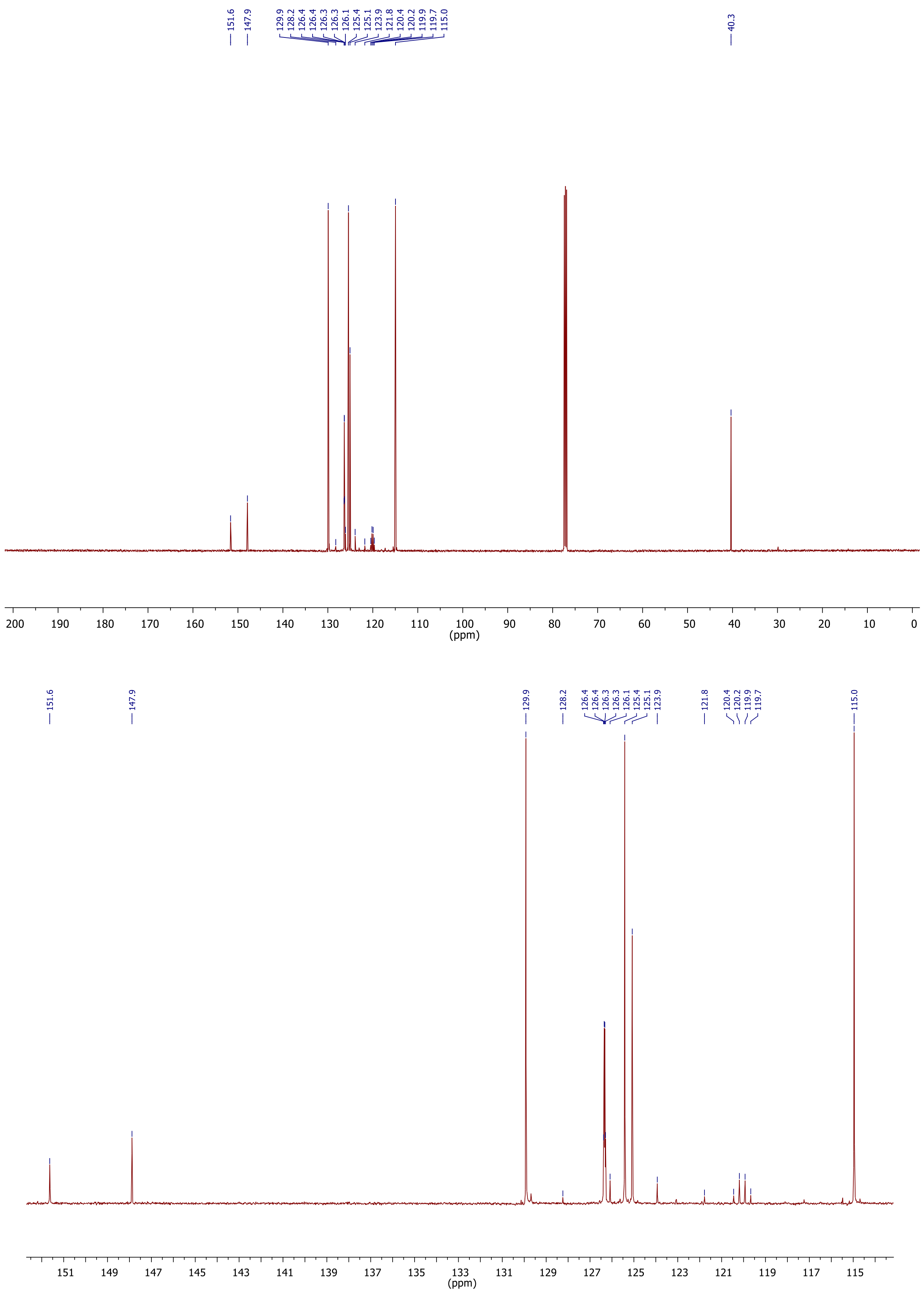

Figure S6b. ^{13}C NMR spectrum of compound **3e** (126 MHz, $CDCl_3$).

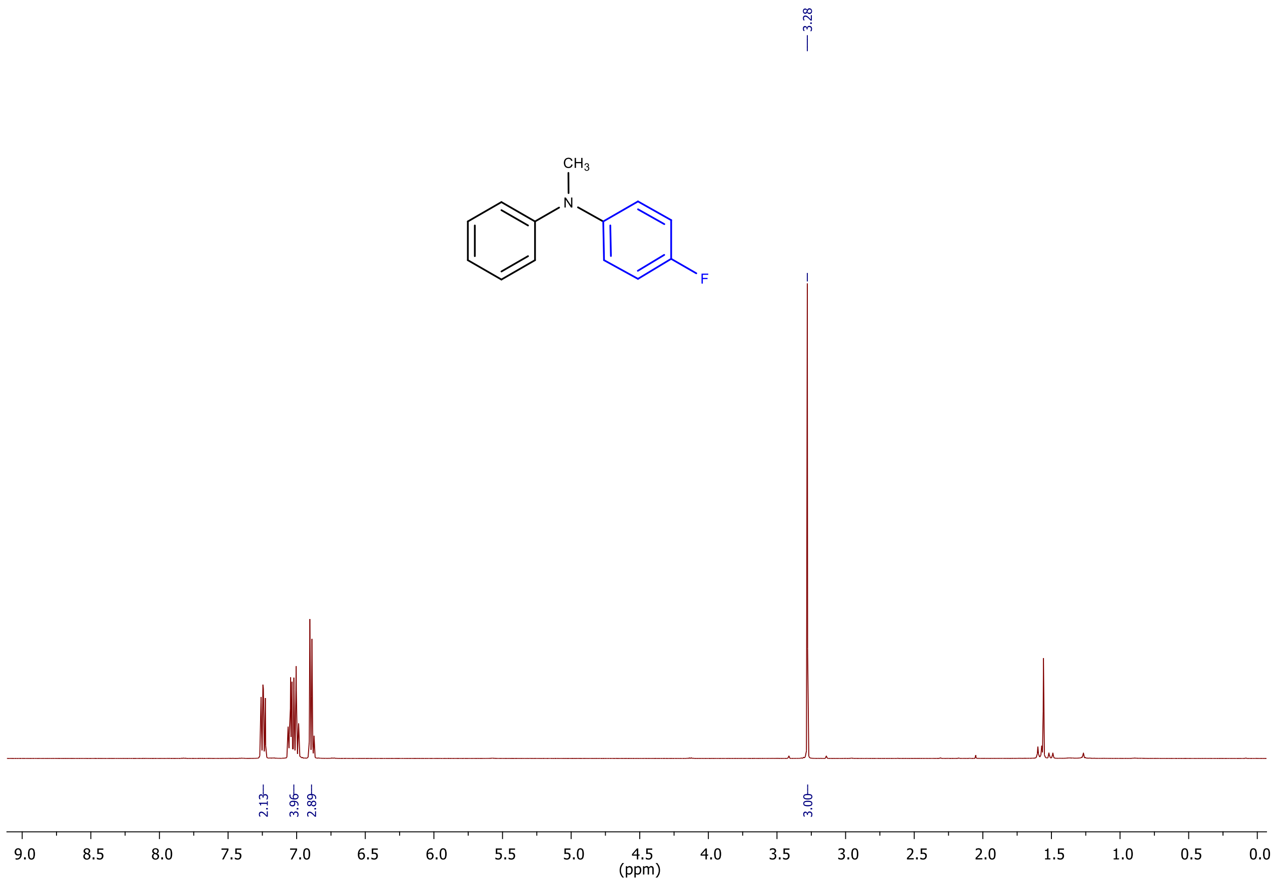

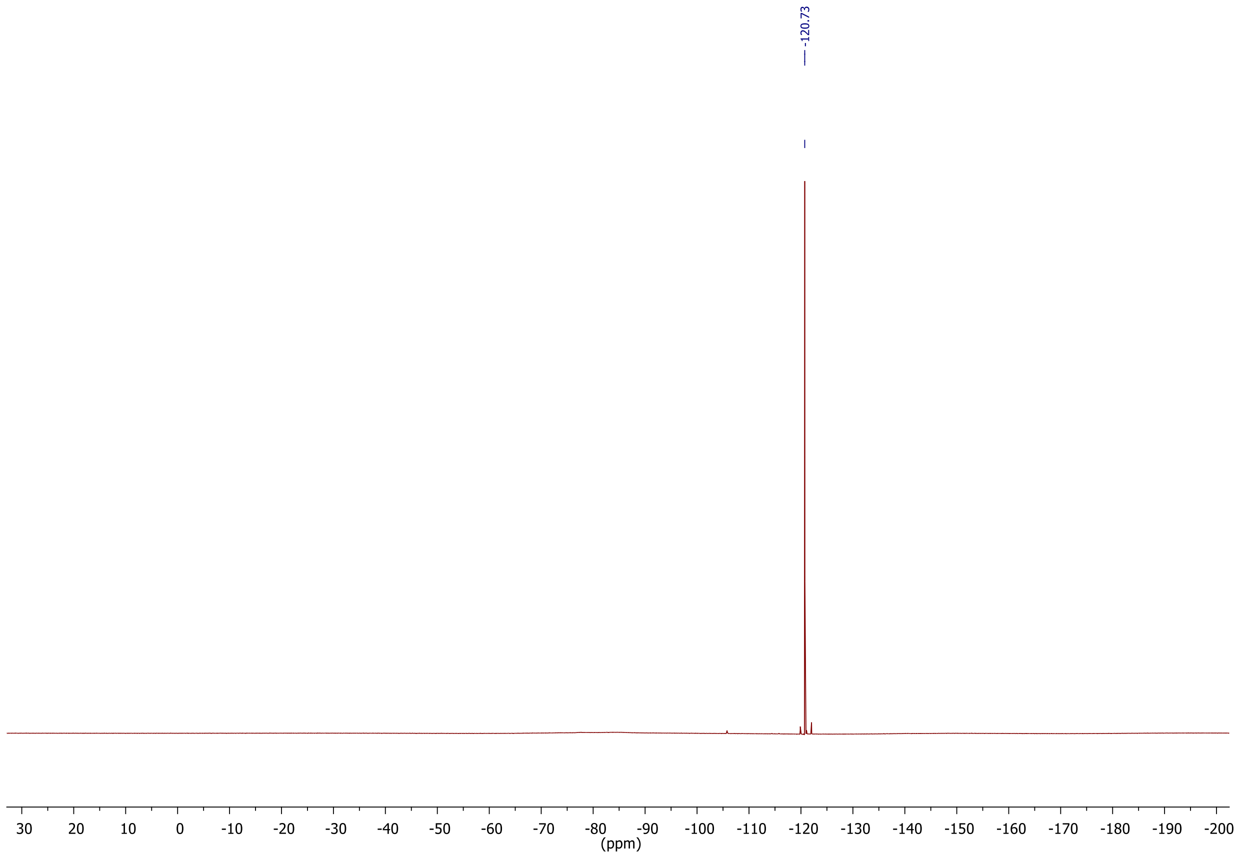

Figure S7a. ^{1}H NMR and ^{19}F NMR spectra of compound **3f** (500 and 470 MHz, $CDCl_3$).

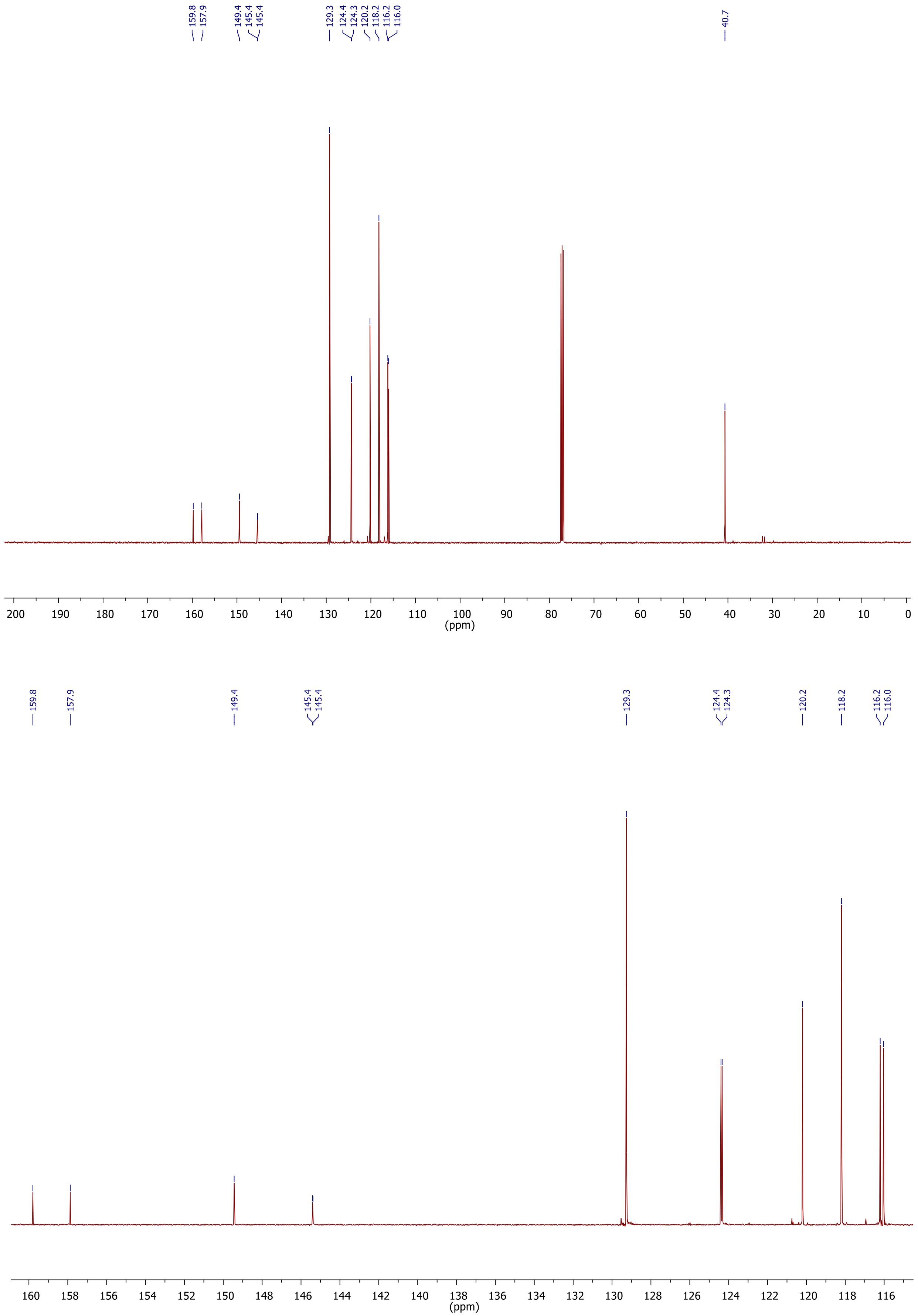

Figure S7b. ^{13}C NMR spectrum of compound **3f** (126 MHz, $CDCl_3$).

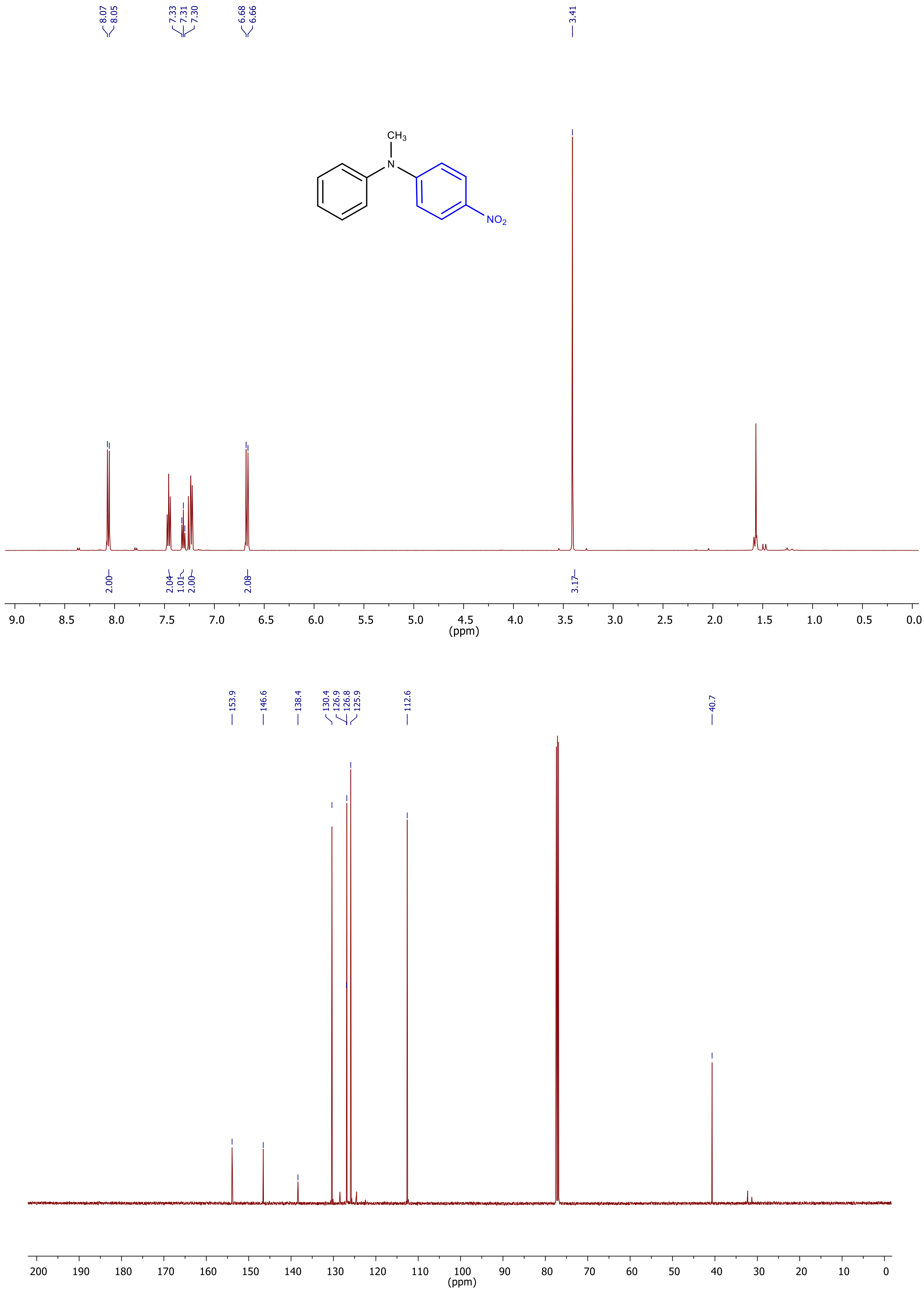

Figure S8. ^{1}H NMR and ^{13}C NMR spectra of compound **3g** (500 and 126 MHz, $CDCl_3$).

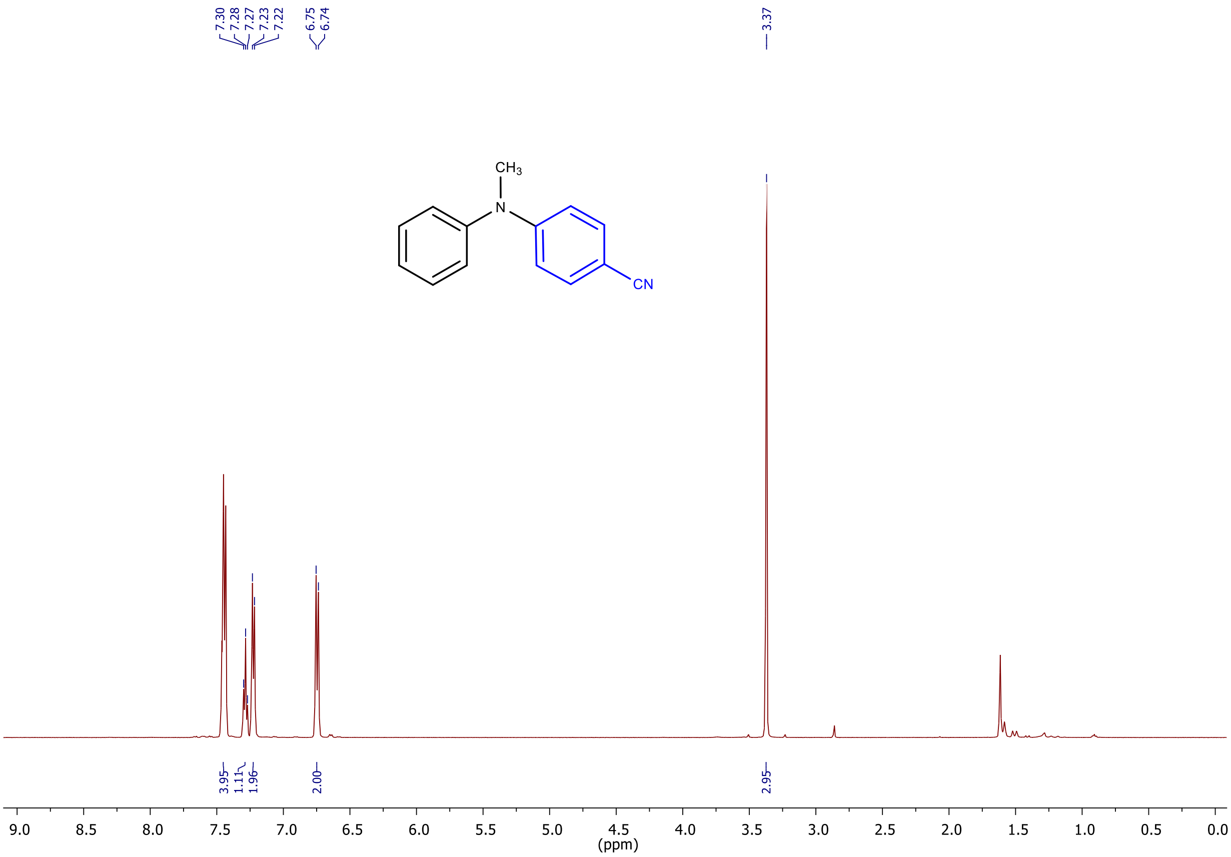

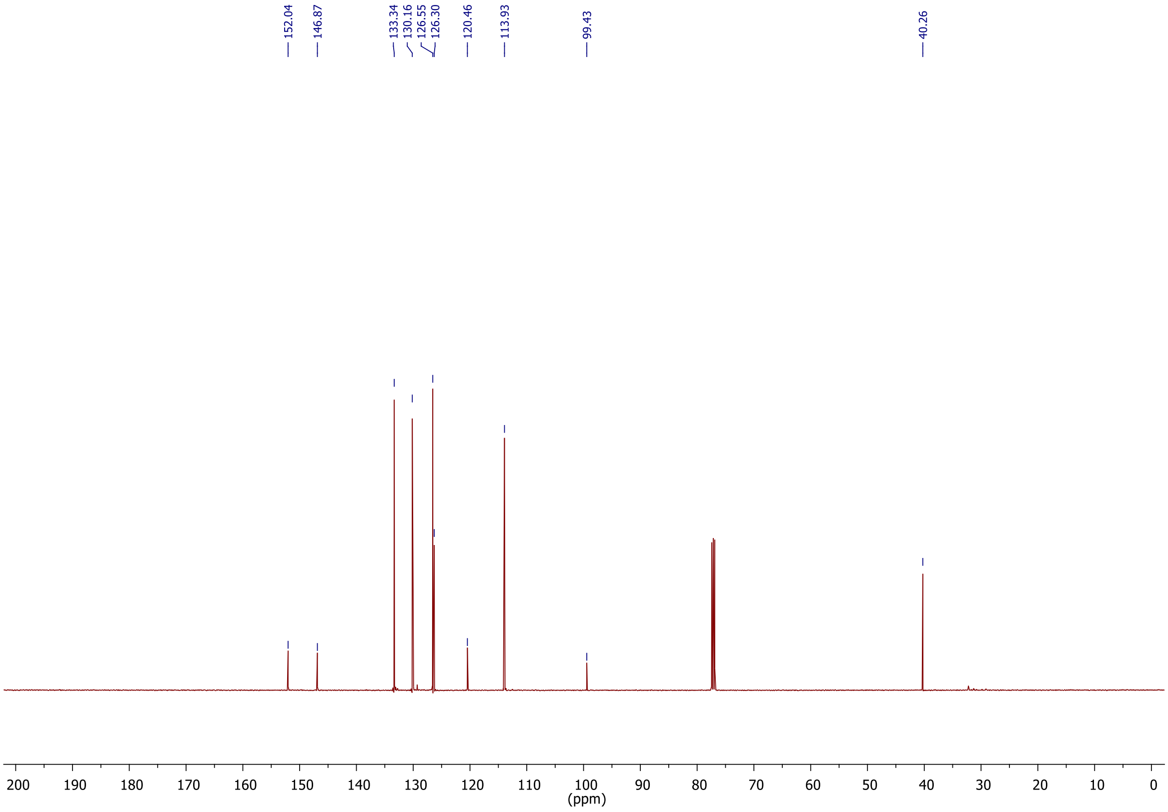

Figure S9. ^{1}H NMR and ^{13}C NMR spectra of compound **3h** (500 and 126 MHz, $CDCl_3$).

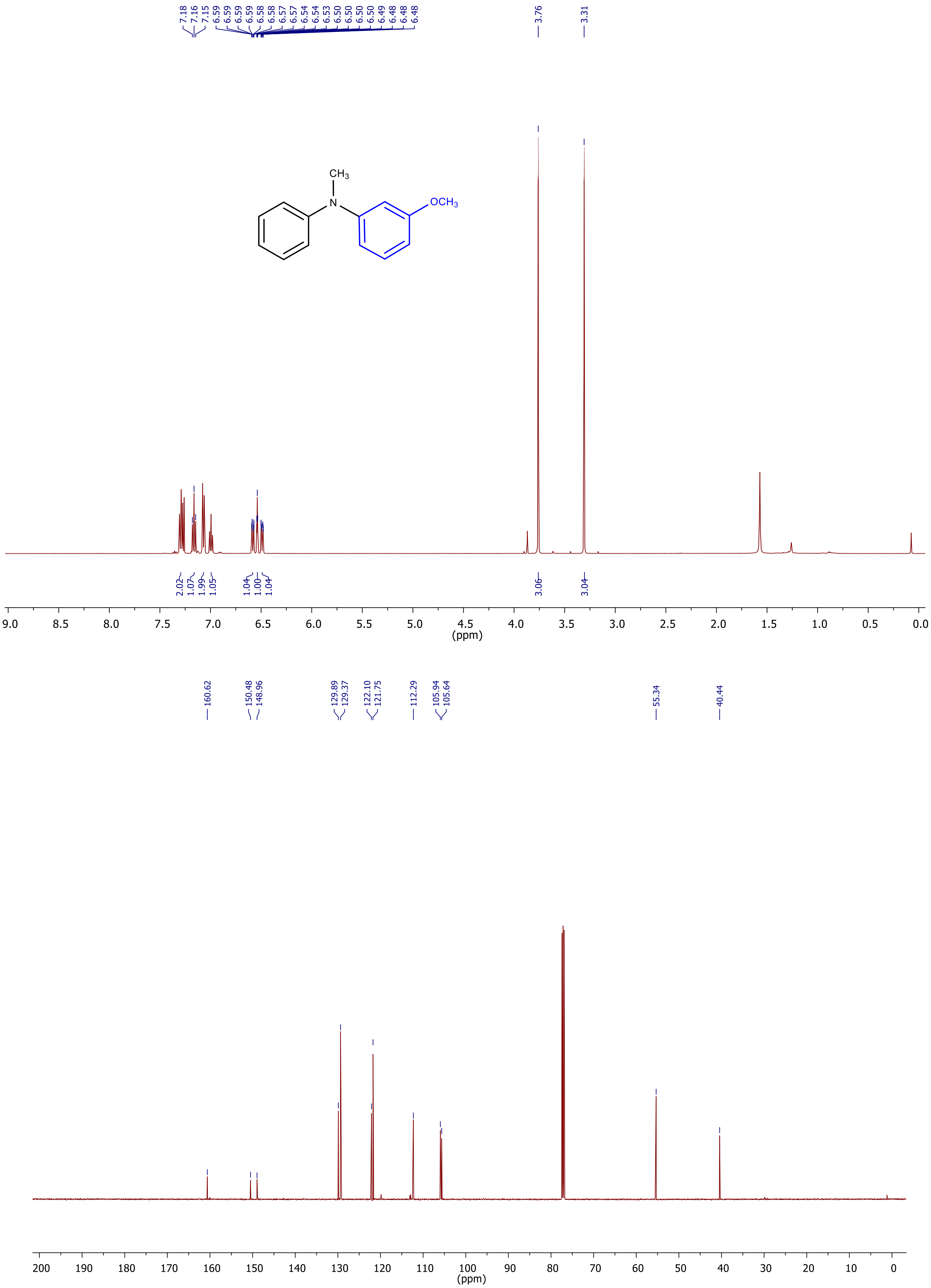

Figure S10. ^{1}H NMR and ^{13}C NMR spectra of compound **3i** (500 and 126 MHz, $CDCl_3$).

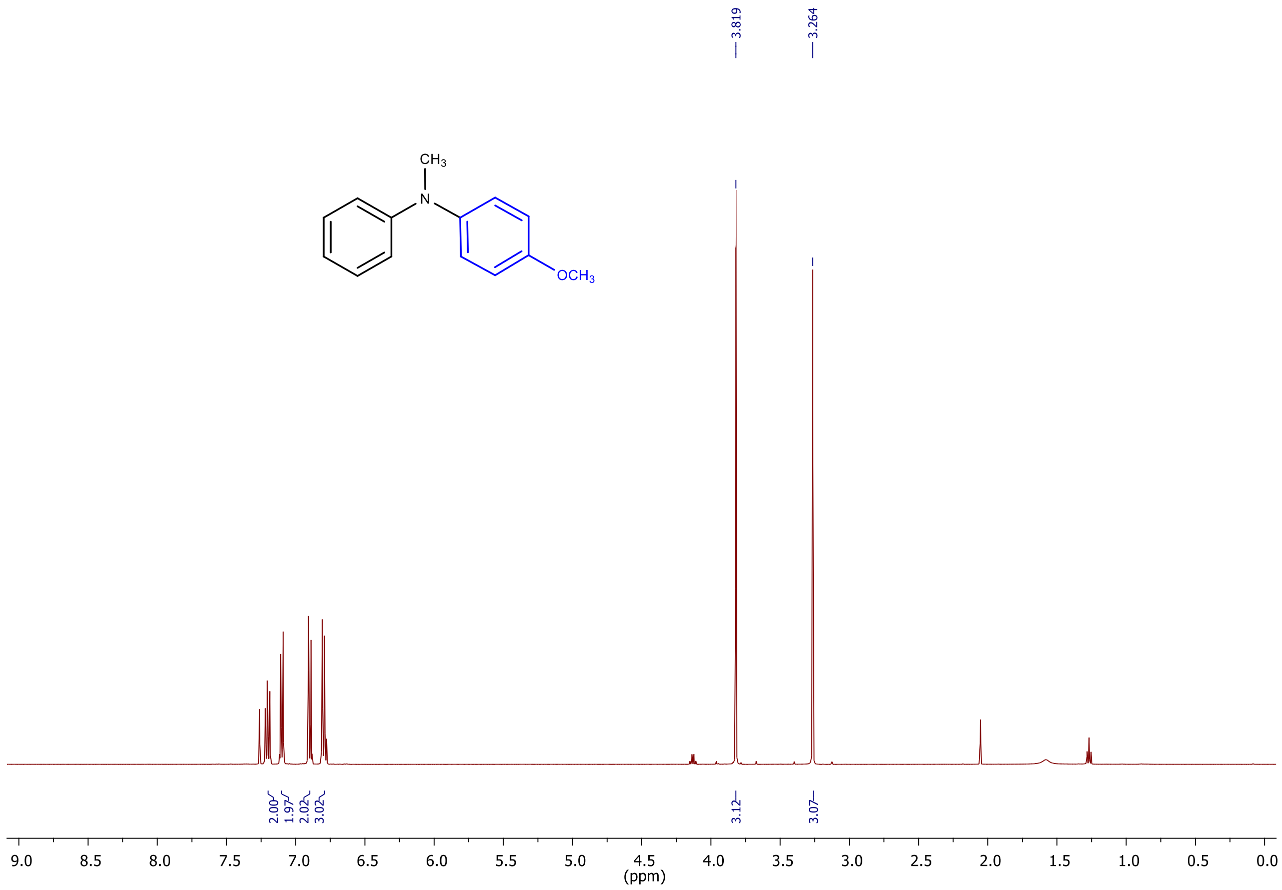

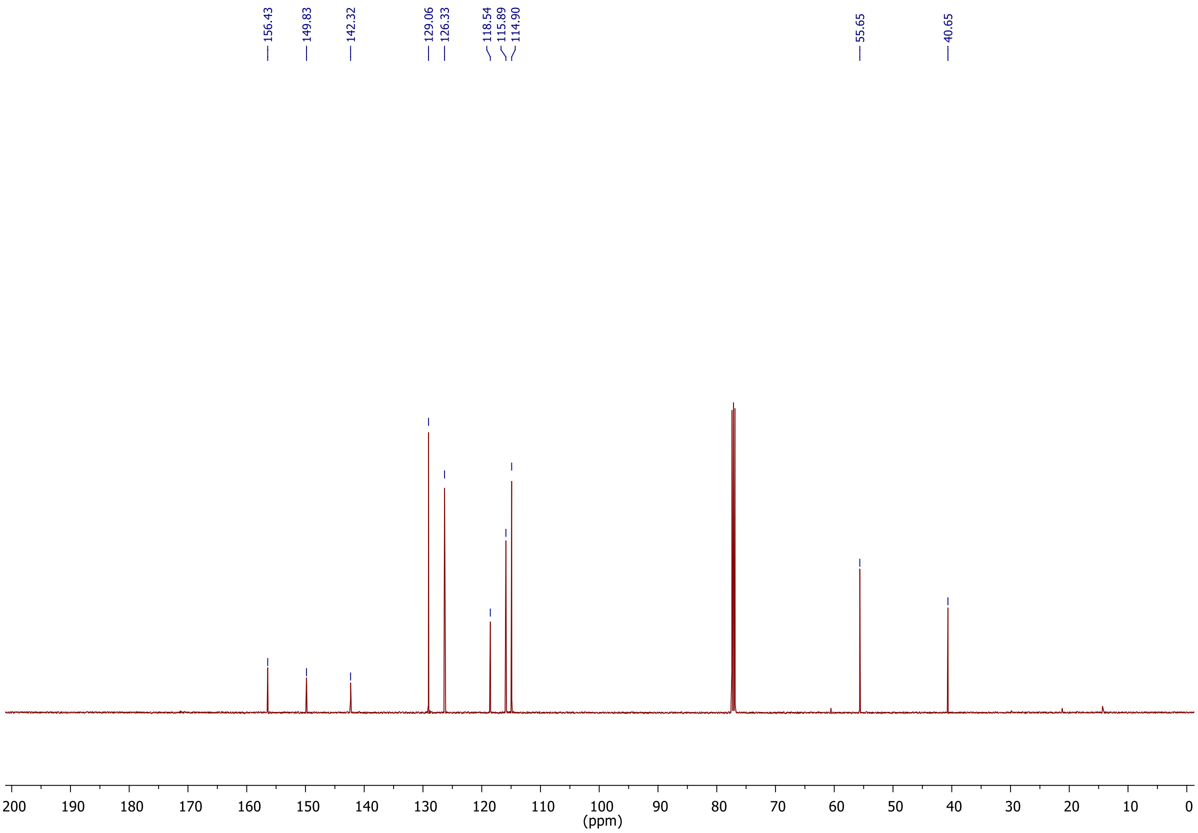

Figure S11. ^{1}H NMR and ^{13}C NMR spectra of compound **3j** (500 and 126 MHz, $CDCl_3$).

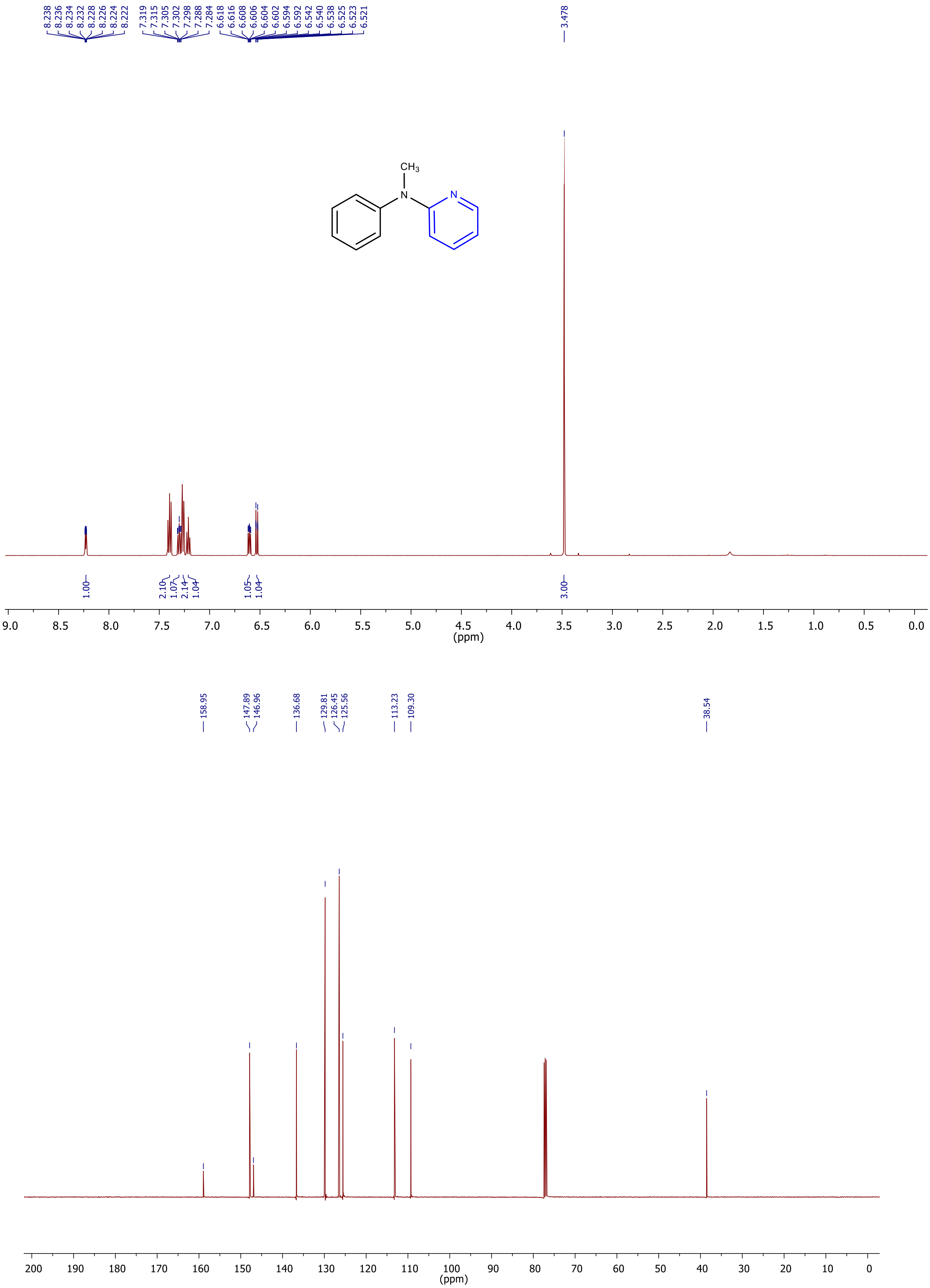

Figure S12. ^{1}H NMR and ^{13}C NMR spectra of compound **3k** (500 and 126 MHz, $CDCl_3$).

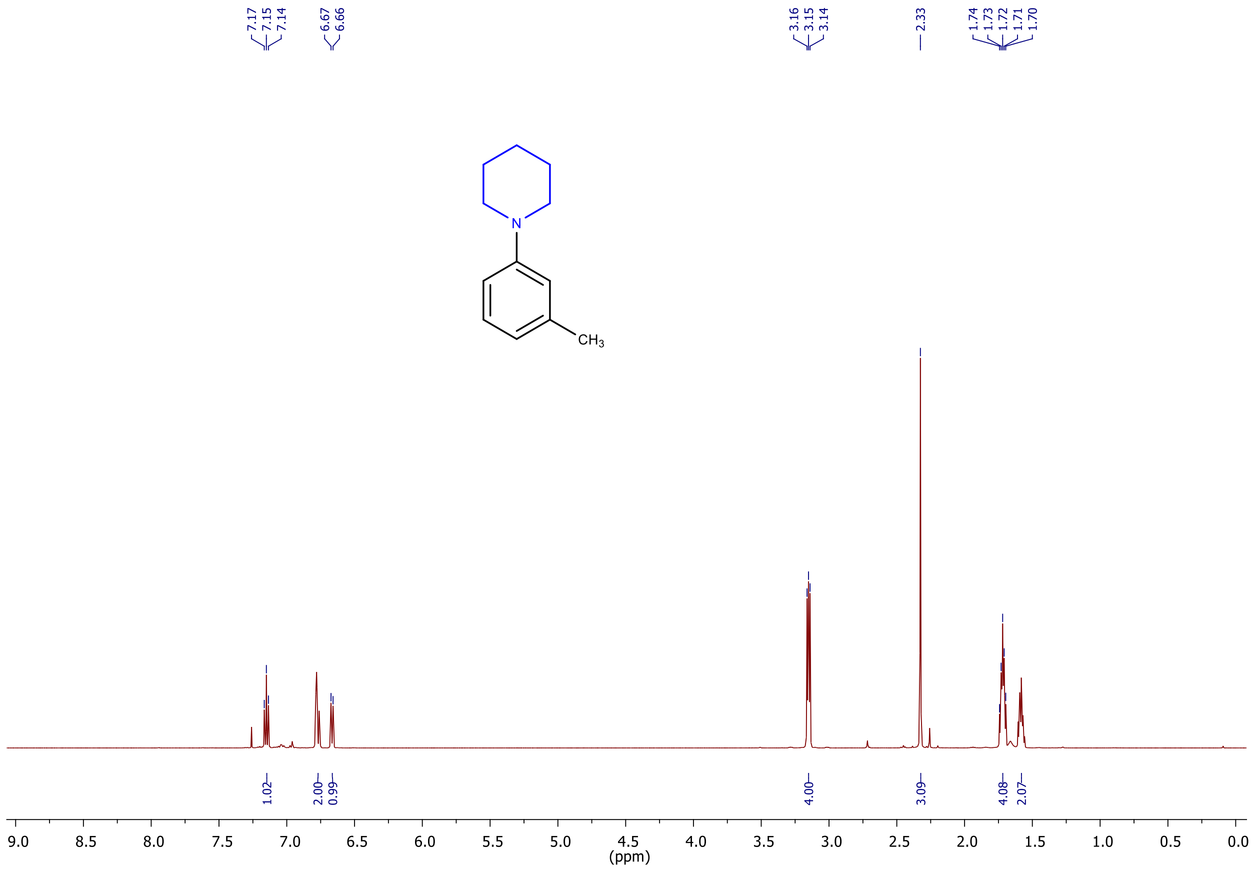

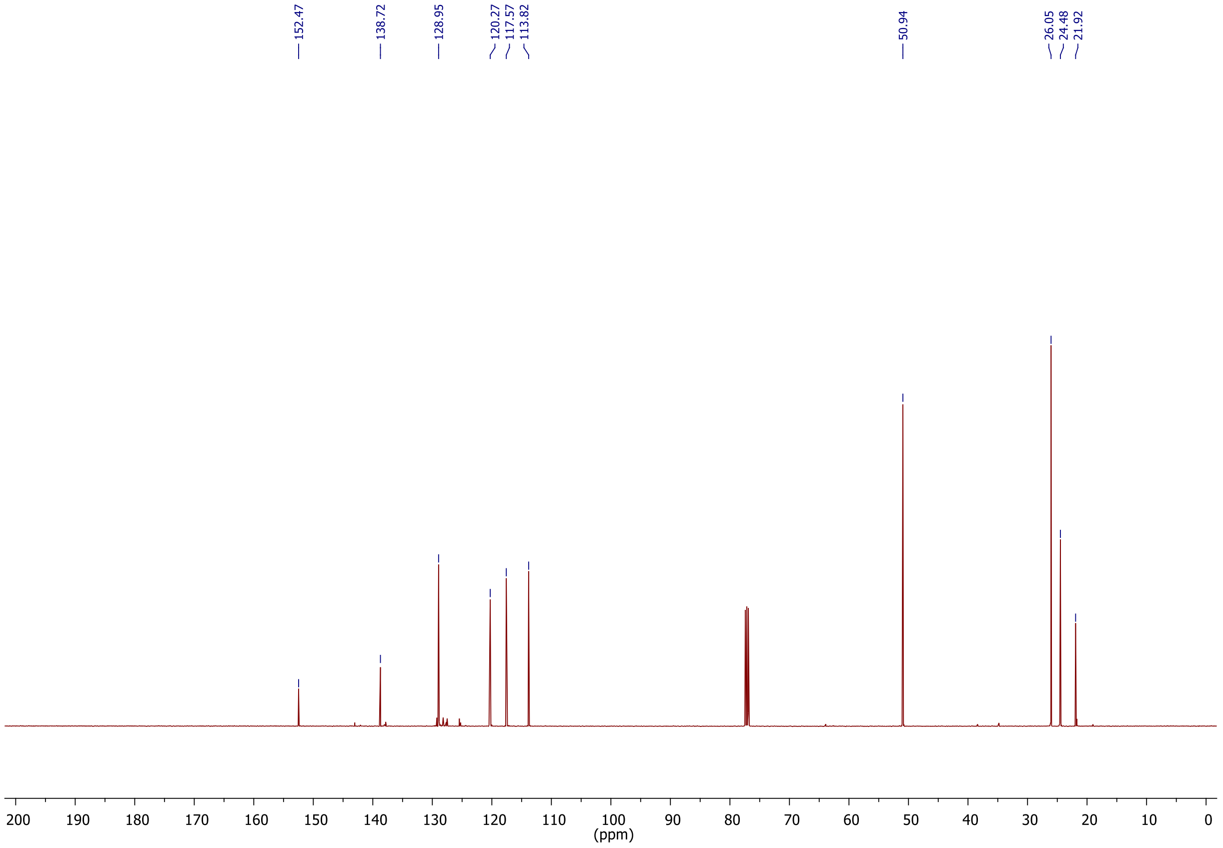

Figure S13. ^{1}H NMR and ^{13}C NMR spectra of compound **3m** (500 and 126 MHz, $CDCl_3$).

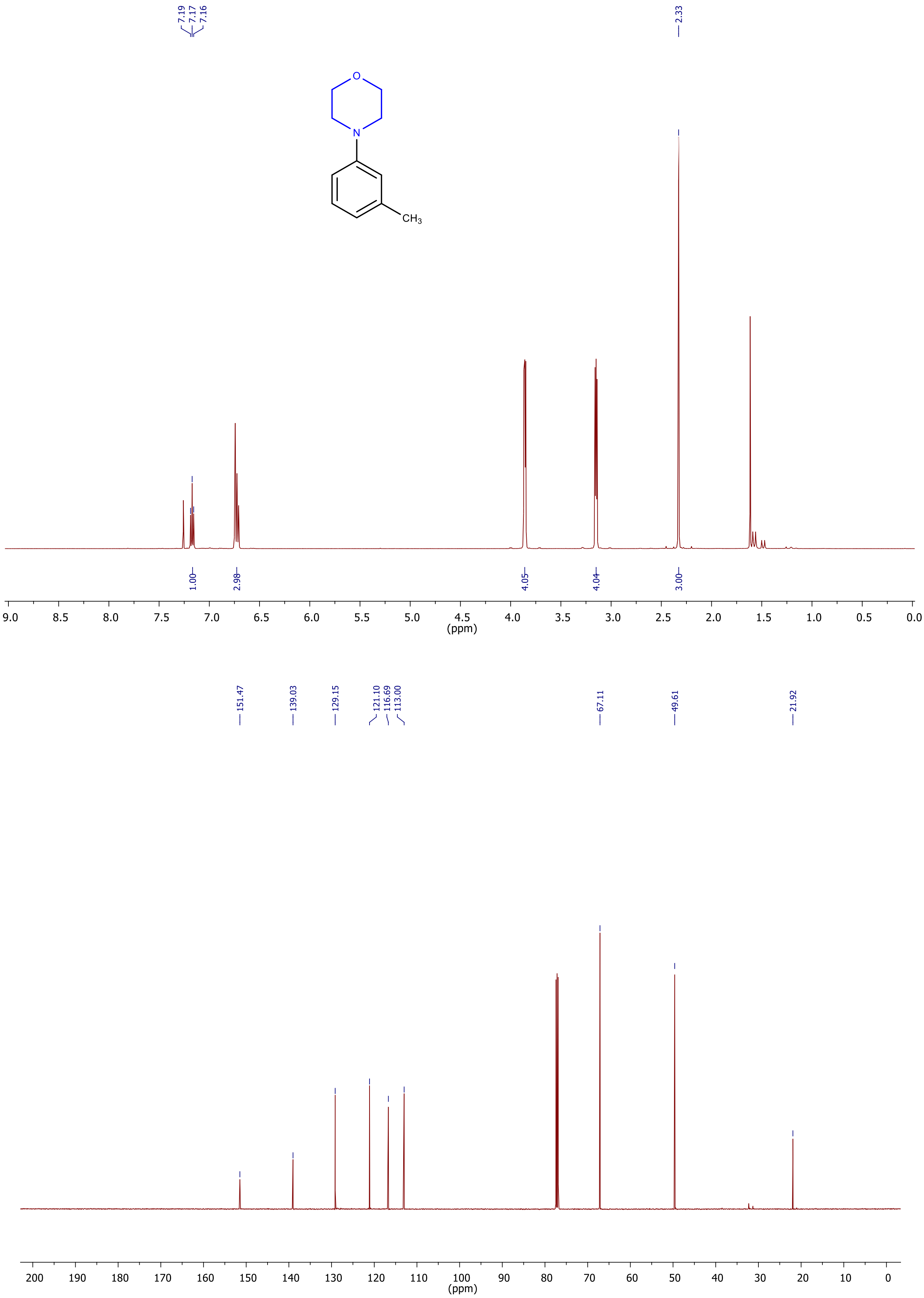

Figure S14. ^{1}H NMR and ^{13}C NMR spectra of compound **3n** (500 and 126 MHz, $CDCl_3$).

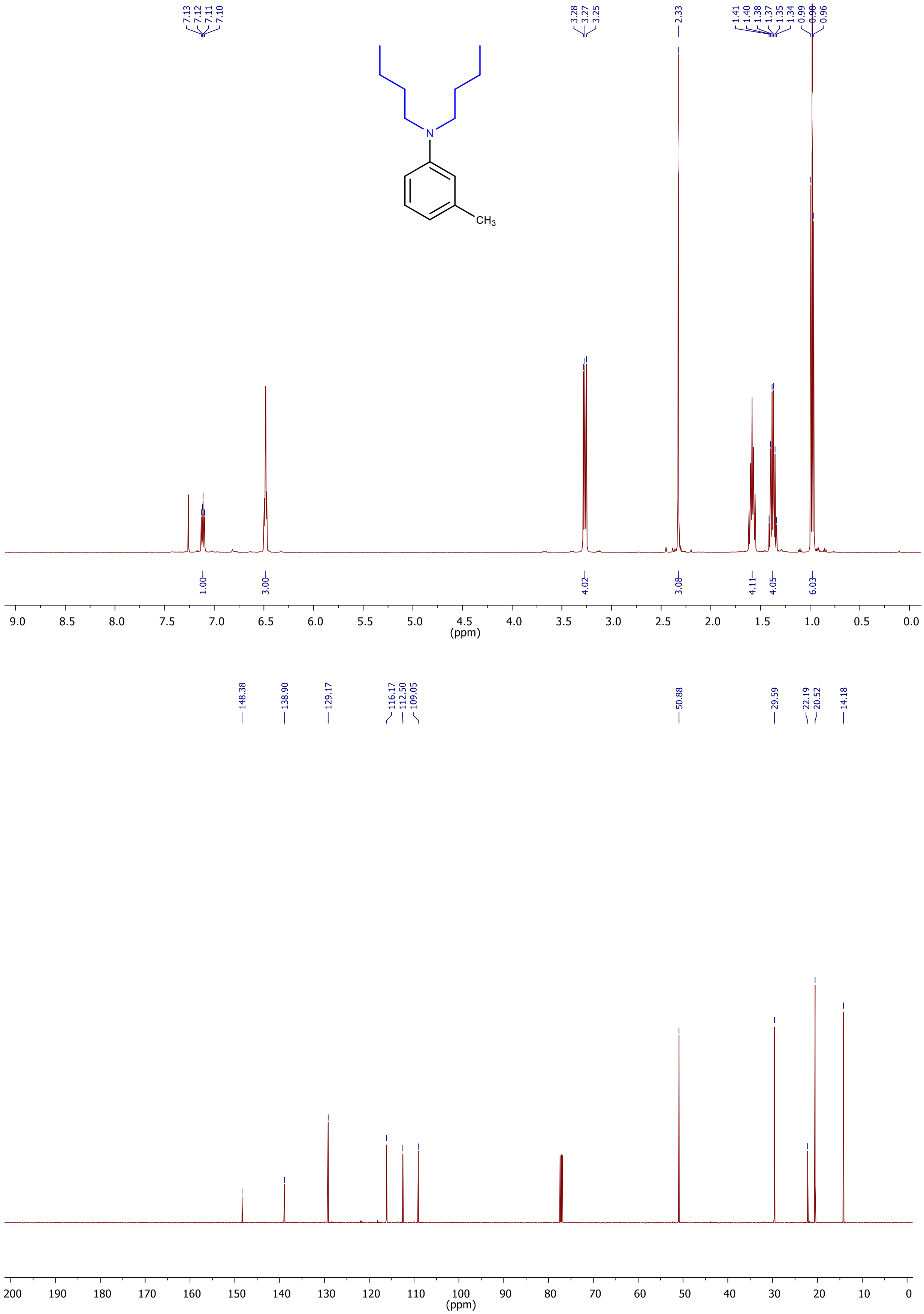

Figure S15. ^{1}H NMR and ^{13}C NMR spectra of compound **3o** (500 and 126 MHz, $CDCl_3$).

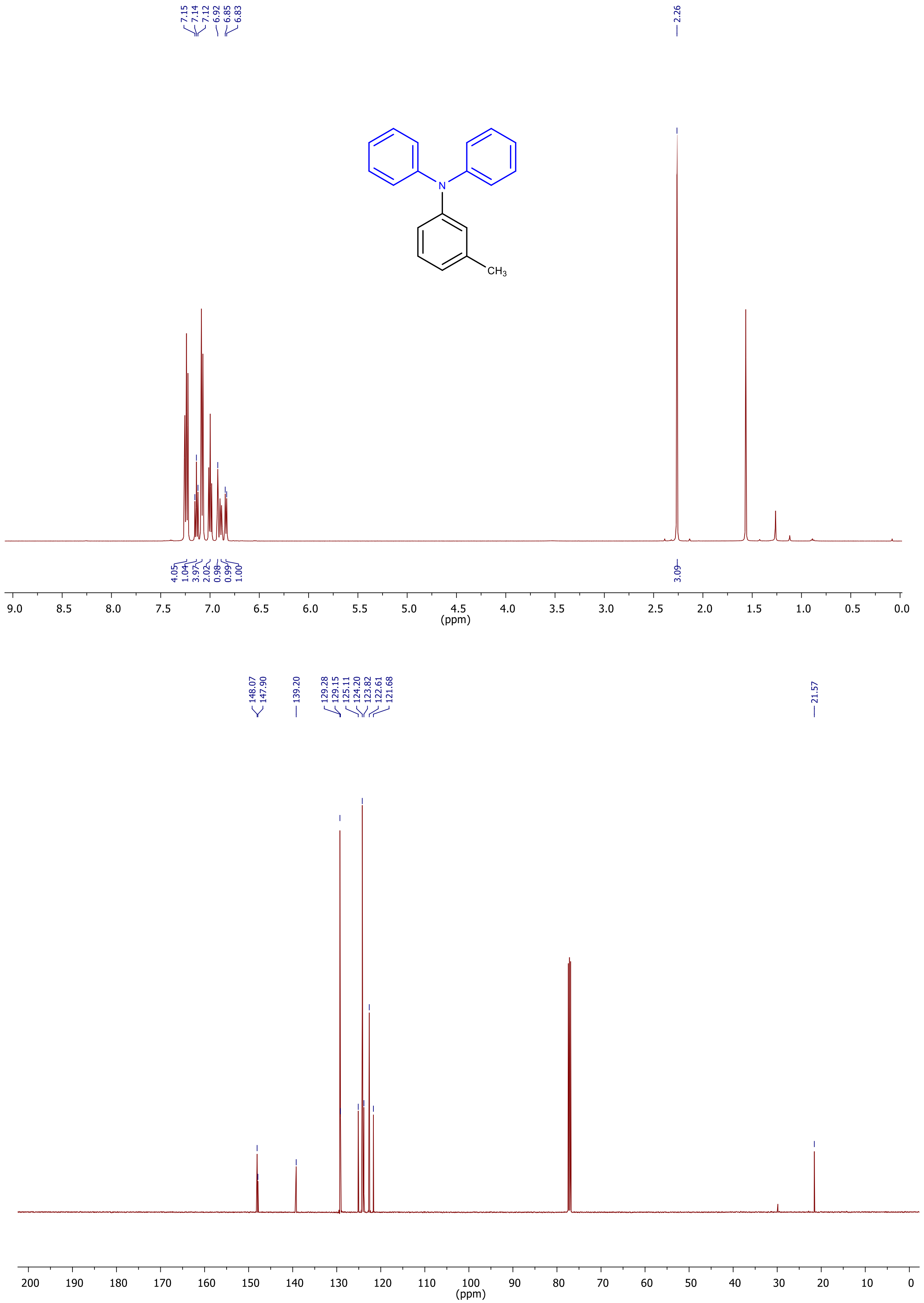

Figure S16. ^{1}H NMR and ^{13}C NMR spectra of compound **3p** (500 and 126 MHz, $CDCl_3$).

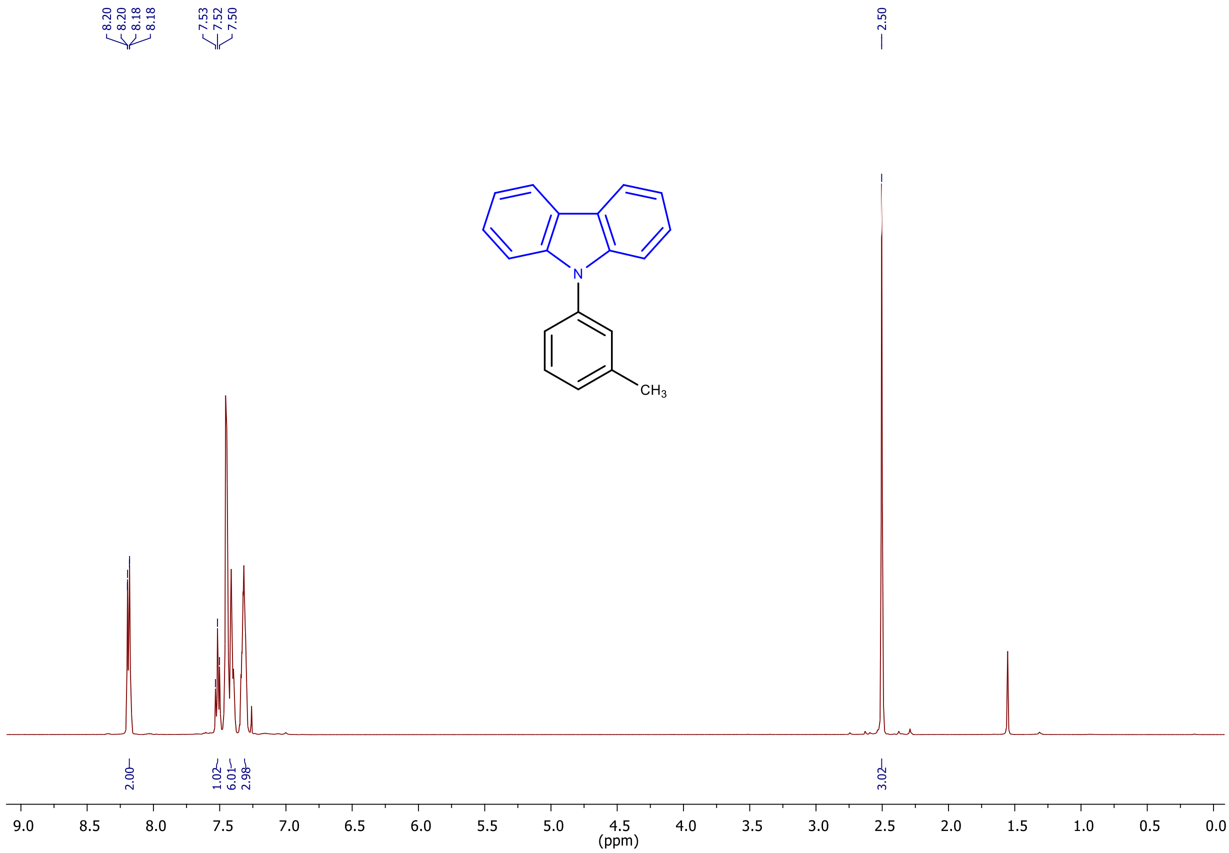

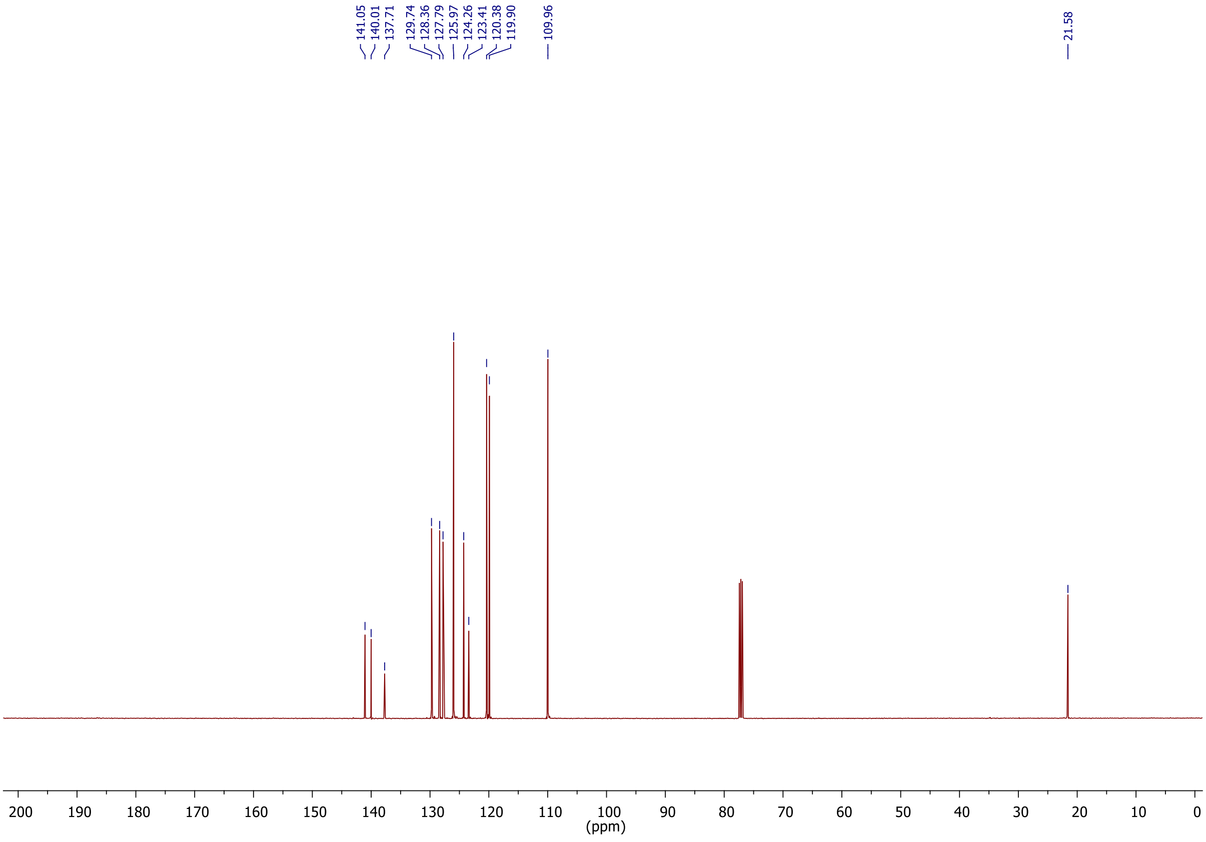

Figure S17. ^{1}H NMR and ^{13}C NMR spectra of compound **3q** (500 and 126 MHz, $CDCl_3$).

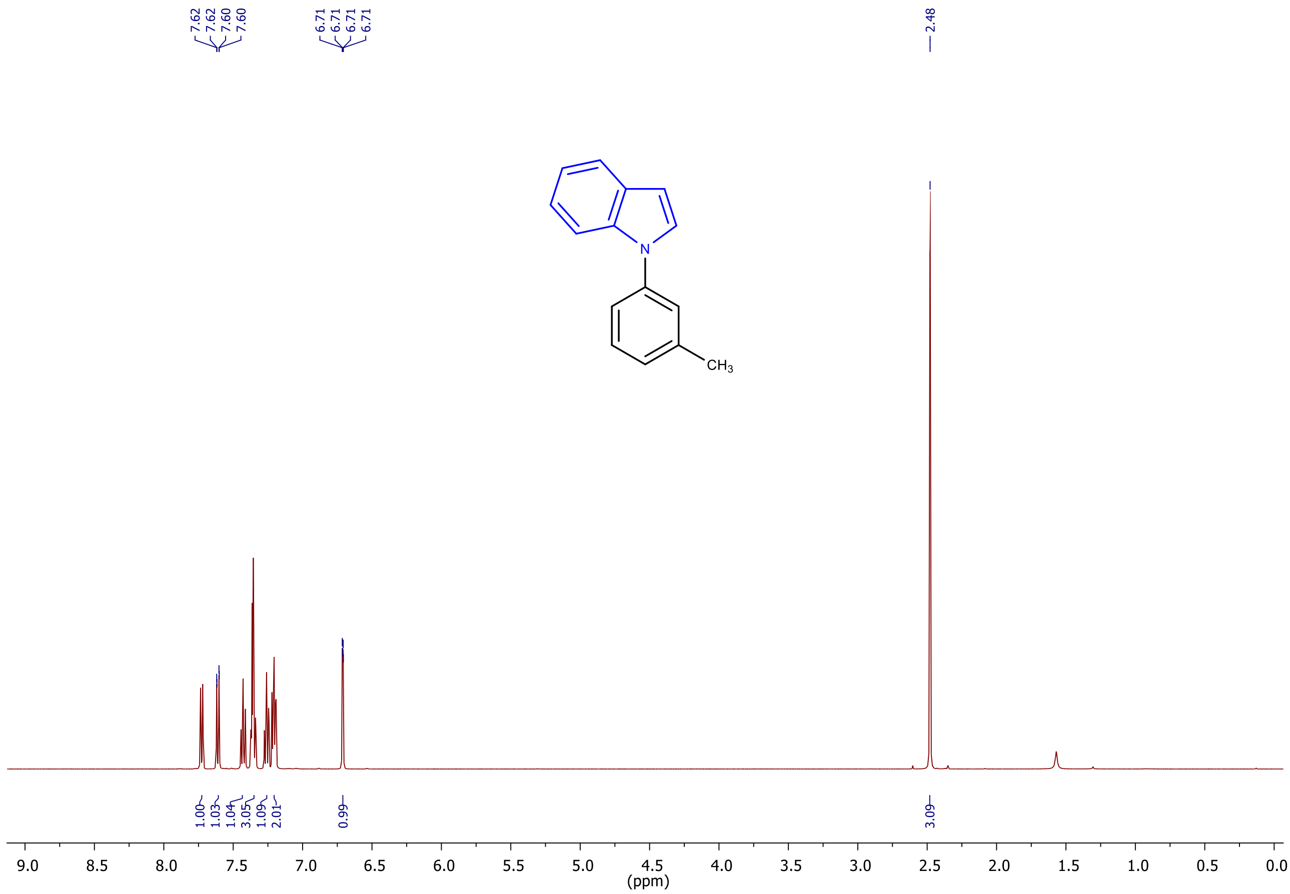

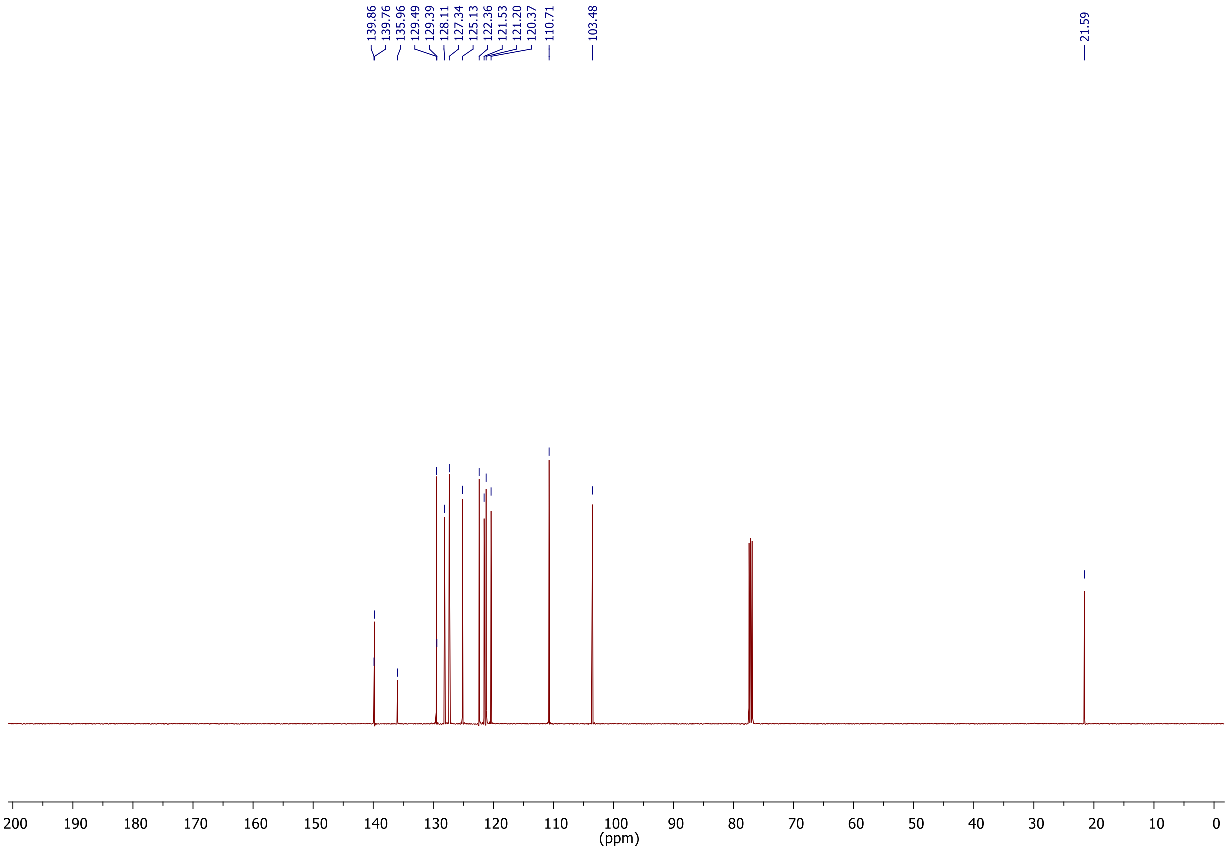

Figure S18. ^{1}H NMR and ^{13}C NMR spectra of compound **3r** (500 and 126 MHz, $CDCl_3$).

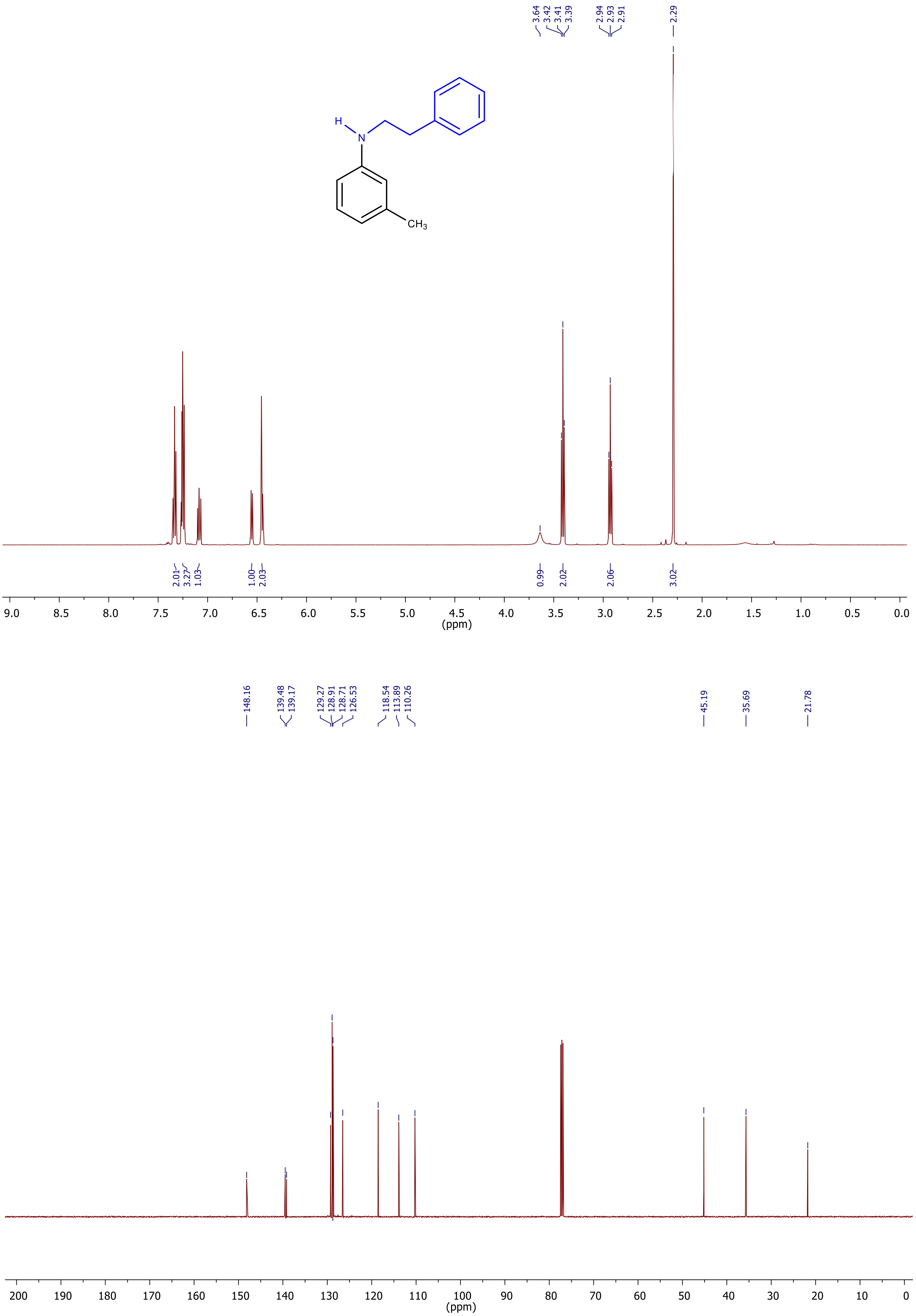

Figure S19. ^{1}H NMR and ^{13}C NMR spectra of compound **3s** (500 and 126 MHz, $CDCl_3$).

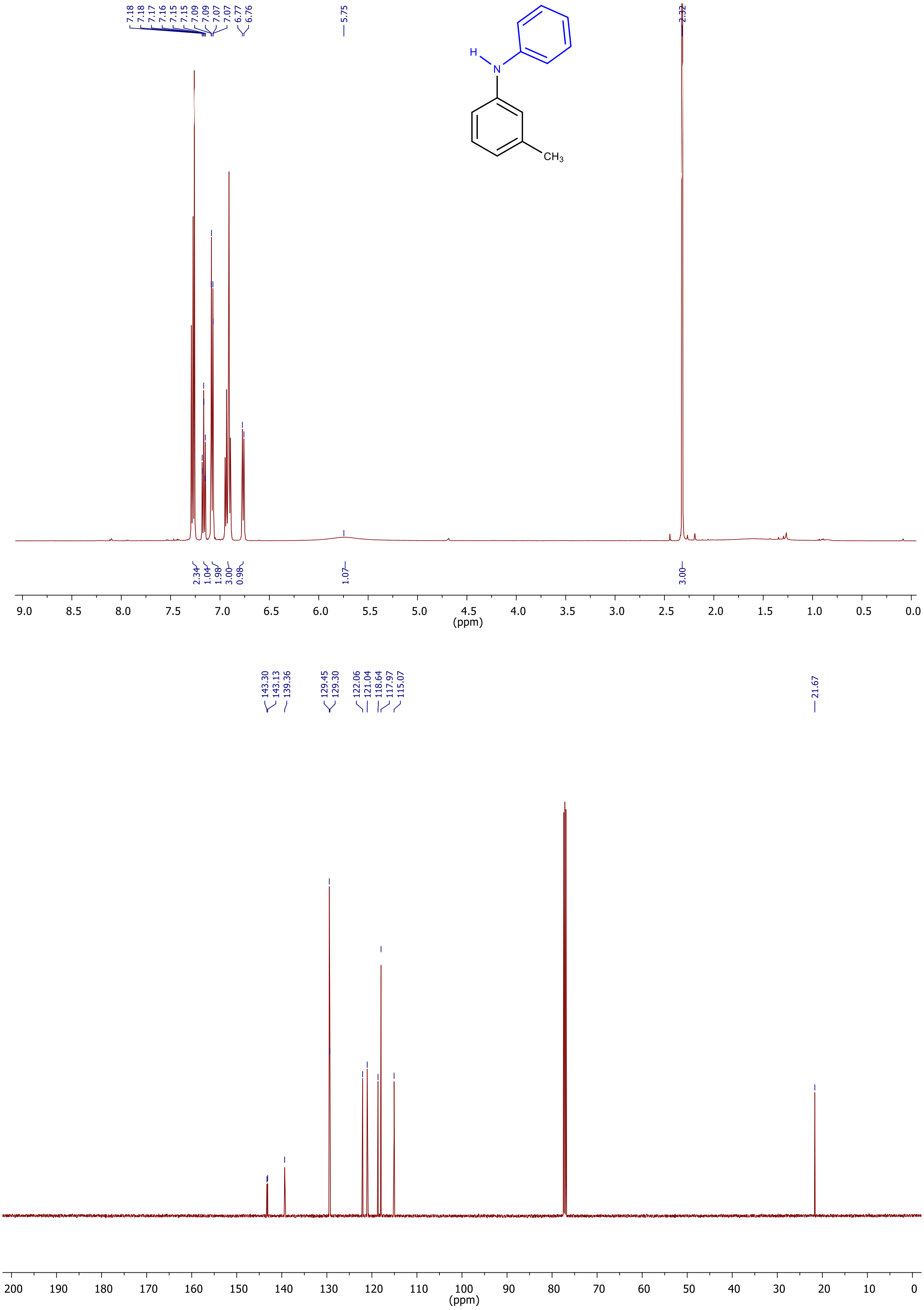

Figure S20. ^{1}H NMR and ^{13}C NMR spectra of compound **3t** (500 and 126 MHz, $CDCl_3$).

Calculation of E-factor values[28]

Complete E-factor (cE) and simple E-factor (sE) formulae:

$$cE = \frac{\Sigma m(\text{Raw materials}) + \Sigma m(\text{Reagents}) + \Sigma m(\text{Solvents}) + \Sigma m(\text{Water}) - m(\text{Product})}{m(\text{Product})}$$

$$sE = \frac{\Sigma m(\text{Raw materials}) + \Sigma m(\text{Reagents}) - m(\text{Product})}{m(\text{Product})}$$

The sE includes the mass of all non-recyclable materials while excluding water and solvents, making it better suited for process route scouting. In contrast, the cE accounts for all process materials, including solvents and water, assuming no recycling, offering a comprehensive assessment of a chemical process's overall efficiency and environmental impact.

*Synthesis of **3a** (10 mmol scale):*

A Carius tube (ø=2 cm) with a screw cap and equipped with a magnetic stirrer was charged with sodium *tert*-butoxide (1.44 g, 15 mmol), Pd(^{t}Bu$_3$P)$_2$ (256 mg, 0.5 mmol) and CMPE (4 mL, 3.40 g, 34 mmol), then *N*-methylaniline (1.07g, 10 mmol) and 3-iodotoluene (3.27g, 15 mmol) were added. The reaction mixture was reacted under IR irradiation and magnetic stirring. After 2 h, the mixture was cooled to room temperature and 2 mL of water was added. The organic phase, after a short percolation on silica gel (0.5 g), was concentrated under vacuum. Then the crude reaction mixture was purified by distillation with Kugelrohr (Büchi Glass Oven B-580) at 25 mbar (glass oven temperature: 200-210°C) leading to 1.644 g of **3a** (83% yield).

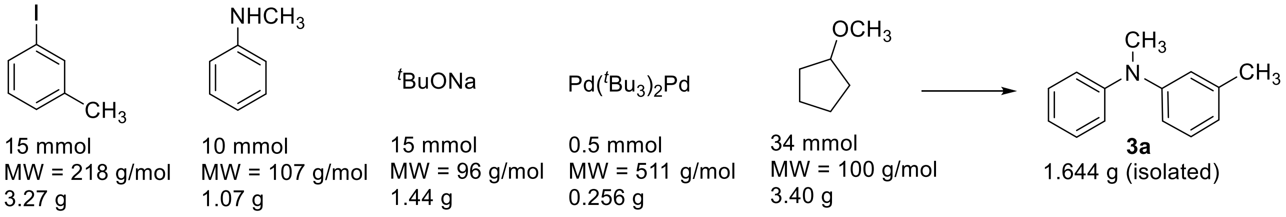

Work-up: H_2O = 2mL (2 g); silica gel = 0.5 g

Distillation (solvent for product manipulation): AcOEt (d = 0.9 g/mL) = 3 mL (2.7 g)

cE = 14.636 g (all materials) – 1.644 g (product)/1.644 g (product) = **7.9**

sE = 6.536 g (all non-recyclable materials) – 1.644 g (product)/1.644 g (product) = **3.0**

Supplementary References

[15] P. Huang, B.-Y. He, H.-M. Wang, J.-M. Lu *Synthesis* **2015**, *47,* 221-227.

[16] P. Weber, T. Scherpf, I. Rodstein, D. Lichte, L. T. Scharf, L. J. Gooßen, V. H. Gessner *Angew. Chem. Int. Ed. Engl.* **2019**, *58*, 3203-3207.

[17] M. A. Topchiy, A. F. Asachenko, M. S. Nechaev *Eur. J. Org. Chem.* **2014**, 3319-3322.

[18] S. M. Wong, P. Y. Choy, Q. Zhao, O. Y. Yuen, C. C. Yeung, C. M. So, F. Y. Kwong *Organometallics* **2021**, *40*, 14, 2265-2271.

[19] S. Meiries, A. Chartoire, A. M. Z. Slawin, S. P. Nolan *Organometallics* **2012**, *31*, 8, 3402-3409.

[20] L. L. Hill, L. R. Moore, Rongcai Huang, R. Craciun, A. J. Vincent, D. A. Dixon, J. Chou, C. J. Woltermann, K. H. Shaughnessy *J. Org. Chem.* **2006**, *71*, 5117-5125.

[21] Z.-M. Zhang, Y.-T. Xu, L.-X. Shao *J. Organomet. Chem.* **2021**, *940*, 121683.

[22] N. Kataoka, Q. Shelby, J. P. Stambuli, J. F. Hartwig *J. Org. Chem.* **2002**, *67*, 5553-5566.

[23] H.-W. Liu, P. He, W.-T. Li, W. Sun, K. Shi, Y.-Q. Wang, Q.-K. Mo, X.-Y. Zhang, S.-F. Zhu *Angew. Chem. Int. Ed.* **2023***, 62,* e202309111.

[24] L. Peng, Y. Zhao, J. Chen, H. Lu, Z. Tang, Y. Chen, S.-F. Yin, N. Kambe, R. Qiu *J. Org. Chem.* **2024**, *89*, 183-190.

[25] Y. Zhanga, Z.-Y. Hua, X.-C. Lia, X.-X. Guo *Synthesis* **2019**, *51*, 1803-1808.

[26] M. T. Barros, S. S. Dey, C. D. Maycock, P. Rodrigues *Tetrahedron* **2012**, *68*, 6263-6268.

[27] S. Yang, X. Yu, A. Poater, L. Cavallo, C. S. J. Cazin, S. P. Nolan, M. Szostak *Org. Lett.* **2022**, 24, 9210-9215.

[28] R. A. Sheldon *Green Chem.* **2017**, *19,* 18-43.
